# Global analysis and prediction of fluoride in groundwater

**DOI:** 10.1038/s41467-022-31940-x

**Published:** 2022-08-01

**Authors:** Joel Podgorski, Michael Berg

**Affiliations:** Department of Water Resources and Drinking Water, Eawag, Swiss Federal Institute of Aquatic Science and Technology, 8600 Dübendorf, Switzerland

**Keywords:** Geochemistry, Water resources, Environmental monitoring

## Abstract

The health of millions of people worldwide is negatively impacted by chronic exposure to elevated concentrations of geogenic fluoride in groundwater. Due to health effects including dental mottling and skeletal fluorosis, the World Health Organization maintains a maximum guideline of 1.5 mg/L in drinking water. As groundwater quality is not regularly tested in many areas, it is often unknown if the water in a given well or spring contains harmful levels of fluoride. Here we present a state-of-the-art global fluoride hazard map based on machine learning and over 400,000 fluoride measurements (10% of which >1.5 mg/L), which is then used to estimate the human population at risk. Hotspots indicated by the groundwater fluoride hazard map include parts of central Australia, western North America, eastern Brazil and many areas of Africa and Asia. Of the approximately 180 million people potentially affected worldwide, most reside in Asia (51–59% of total) and Africa (37–46% of total), with the latter representing 6.5% of the continent’s population. Africa also contains 14 of the top 20 affected countries in terms of population at risk. We also illuminate and discuss the key globally relevant hydrochemical and environmental factors related to fluoride accumulation.

## Abstract (web summary)

A global fluoride hazard prediction map was created using machine learning and over 400,000 fluoride measurements, this shows ~180 million people are potentially affected by chronic fluoride exposure worldwide, mostly in Asia and Africa.

## Introduction

The natural occurrence of high concentrations of fluoride in groundwater is a global health concern potentially affecting 100’s of millions of people, predominantly in the Global South^1–14^. The health effects resulting from the long-term ingestion of fluoride include dental and skeletal fluorosis, in many cases severely impacting the lives of those affected^15–21^. Abundant in Earth’s crust, the element fluorine forms fluoride (F^−^) minerals found naturally in soil and aquifer sediments that can lead to fluoride accumulation in freshwater resources, particularly groundwater^15,22^. As such, fluoride intake in the human diet comes primarily through food and drinking water. Fluoride has not been proven to be an essential element in the diet, and although moderate concentrations of fluoride can help prevent dental caries (and is therefore often added to toothpaste), concentrations >1.5 mg/L are known to cause dental fluorosis and, at fluoride intake >6 mg/day, crippling skeletal fluorosis^15^. In order to protect against these ailments, the World Health Organization (WHO) maintains a fluoride guideline of 1.5 mg/L for drinking water^23,24^ However, the WHO recommends that national standards take account of the overall exposure to fluoride and set a lower standard if the intake from all sources reaches 6 mg/day. For example, the desirable maximum concentration of fluoride in drinking water (with higher enforceable limits) recommended by India is 1.0 mg/L^25^, whereas that in the U.S. is 2.0 mg/L^26^.

High fluoride concentrations are often found naturally in aquifers in acidic igneous basement rocks, volcanic and geothermal rocks as well as derived sedimentary deposits and metamorphic rocks with high pH and alkalinity, low calcium concentrations, higher temperatures, and/or long groundwater residence times^9,27,28^. High pH promotes the desorption of fluoride from clay; hydroxyl anions (OH^−^) exchange with F^−^ in F-bearing minerals; and bicarbonate (HCO_3_^−^) reacts with fluorite (CaF_2_) to release fluoride, though dissolved calcium can bind with fluoride and remove it from dissolution to again form fluorite^9,29–31^. Furthermore, higher temperatures (e.g., geothermal waters) enhance chemical weathering^31^ and longer groundwater residence times provide more time for reactions to take place^9^. Arid and semi-arid regions are generally more likely to contain high fluoride groundwaters on account of higher pH and alkalinity as well as longer residence times^2,14^. In addition, anthropogenic activities can result in further fluoride input into groundwater, e.g., application of fertilizers, coal combustion, and subsequent rainfall^22^ as well as managed aquifer recharge^32^.

Since fluoride is odorless, tasteless, and transparent, its presence in a groundwater source can remain undetected until the source is eventually tested^33,34^. However, the quality of groundwater in general and the concentration of fluoride, in particular, are often not analyzed in many areas around the world. In order to determine where high concentrations of fluoride are likely to be found and thereby accelerate their rate of discovery, high-resolution geospatial prediction maps can be created^35–37^ that take advantage of known fluoride concentrations and the natural conditions related to fluoride accumulation in groundwater^38–40^. This can be done using machine-learning approaches that establish statistical relationships between environmental parameters and known contaminant concentrations^37–39^. The resulting model is then applied to the environmental variables to generate a prediction map.

Here we assemble an unprecedentedly large dataset of groundwater fluoride concentrations to develop a global prediction map of the occurrence of fluoride in groundwater exceeding the WHO guideline concentration of 1.5 mg/L. For this purpose, we apply a random-forest machine-learning algorithm and the latest available global datasets of relevant environmental parameters. This model and resulting map provide the most detailed assessment yet available of the global extent of fluoride contamination, allowing for the identification of hotspots and less vulnerable regions as well as the populations most affected. Furthermore, we take advantage of the great number of groundwater fluoride data points to evaluate the environmental and hydrochemical factors related to the geogenic (natural) occurrence of fluoride at a global scale.

## Results

### Global prediction model of fluoride in groundwater

**Fig. 1:**
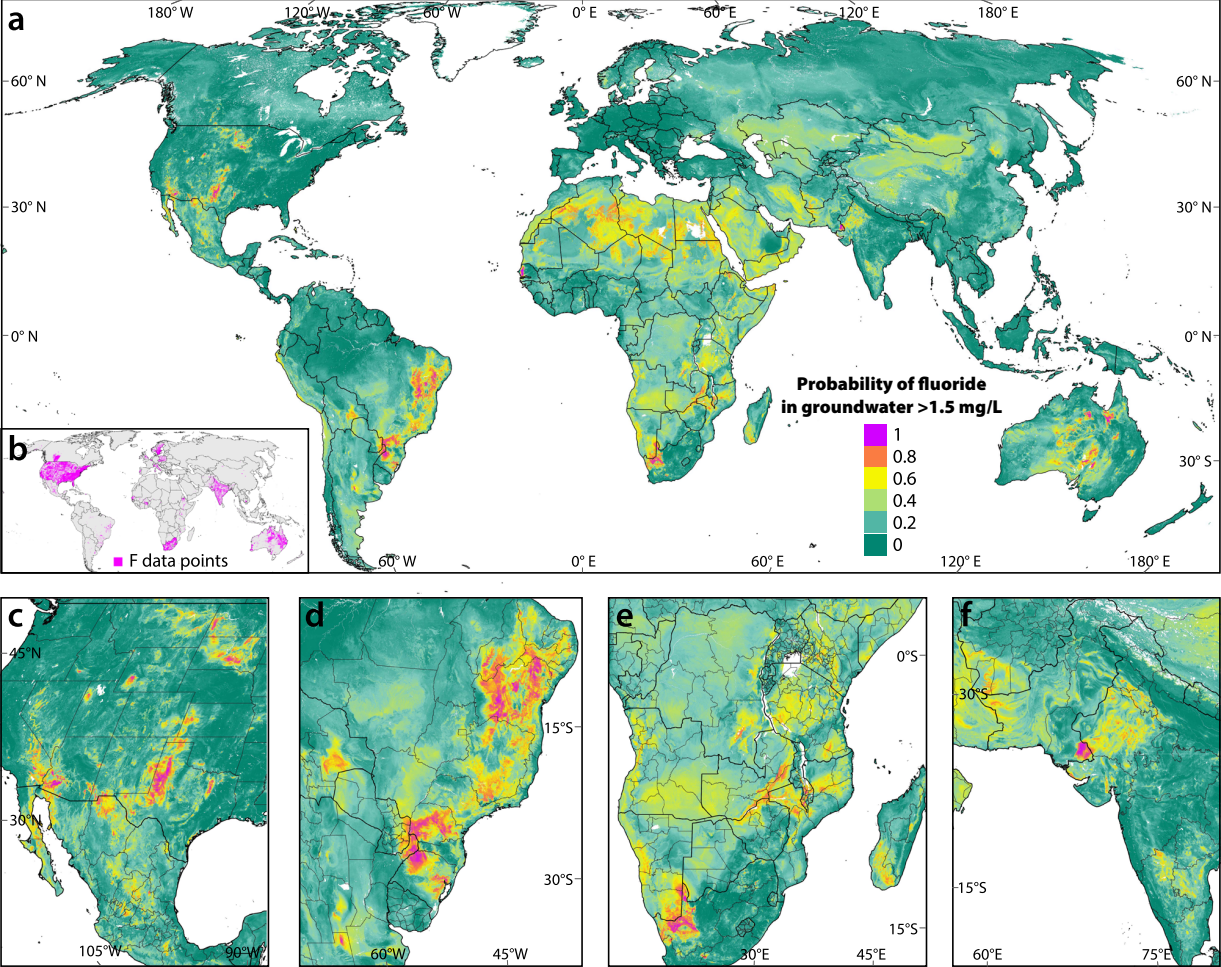
Fluoride in groundwater. **a** Probability of naturally occurring fluoride in groundwater exceeding the WHO drinking water guideline of 1.5 mg/L. The map was developed by applying the final random forest model to the 12 most statistically important predictor variables. Panel **b** shows the fluoride data points (*n* = 402,452) used in analysis and modeling. Closer views of the global map are given for the western U.S. and Mexico (**c**), eastern South America (**d**), the southern half of Africa (**e**), and western South Asia (**f**). The data sources are listed in Supplementary Table 1 and a large-scale map of the fluoride points is shown in Supplementary Fig. 1 along with large-scale versions of the prediction map focused on each continent in Supplementary Figs. 3–8.

The random forest machine learning method^41^ was used to model over 400,000 fluoride measurements assembled from 77 countries (Fig. 1b, Supplementary Figs. 1–2, and Supplementary Table 1). This dataset has a mean, median and interquartile concentration range (IQR) of 0.97, 0.30, and 0.10–0.70 mg/L, respectively, and a prevalence of 10.2% of measurements above 1.5 mg/L. For the geospatial model, the twelve statistically most important (as measured by the Gini index) spatially continuous predictor variables were selected using recursive feature elimination (RFE) from an initial set of 62 variables of geology, soil properties, climate, and topography (Supplementary Table 2). All six of the climate variables (1 km resolution) were selected as well as three topographic and two soil parameters (250 m resolution; Supplementary Table 2). The categorical variable of acidic igneous rocks (250 m resolution) was also included based on its clear association with fluoride^2,9,42^. (The inclusion of further geology variables had no appreciable effect on the model.) The minimum number of samples per node was tuned to 1.

By applying the geospatial random forest model to the predictor datasets, a global fluoride prediction map with a pixel-size of 250 m (the higher resolution of the predictors) was created (Fig. 1; more detailed views in Supplementary Figs. 3–8). The map identifies Africa as being particularly exposed to a considerably greater fluoride hazard than all other continents. That is, in 15% of the area of Africa, there is a greater than 50% probability that fluoride concentrations in groundwater exceed 1.5 mg/L (Supplementary Table 3). The continents with the next highest proportions of high-fluoride areas are Australia/Oceania and South America (each 8%), followed by 2% for Asia and North America and less than 1% for Europe.

The final prediction model was verified by averaging 100 individual random forest models, each using random subsets of 80% of the data for training and the remaining 20% for testing. Overall, the model performs very well, as evidenced by a balanced accuracy of 0.82, a kappa of 0.64, and an AUC of 0.90 (Supplementary Table 4; see “Methods” for descriptions), which is generally superior to those achieved in comparable geospatial models of groundwater quality^36,43–45^. Although all of the model predictors are parameters determined at Earth’s surface (i.e., not over depth), the balanced accuracy stays between 82 and 84% for test samples originating from depths of 0–600 m and drops only to 78% for samples from greater depths (Supplementary Table 4). Most of the available well depths (83%) associated with fluoride measurements fall within the range of 0–600 m, with their mean, median and interquartile range being approximately 306, 70, and 311 m, respectively (Supplementary Table 2). For further comparison, the mean model probabilities, accuracy, and AUC are listed by continent in Supplementary Table 5.

**Fig. 2:**
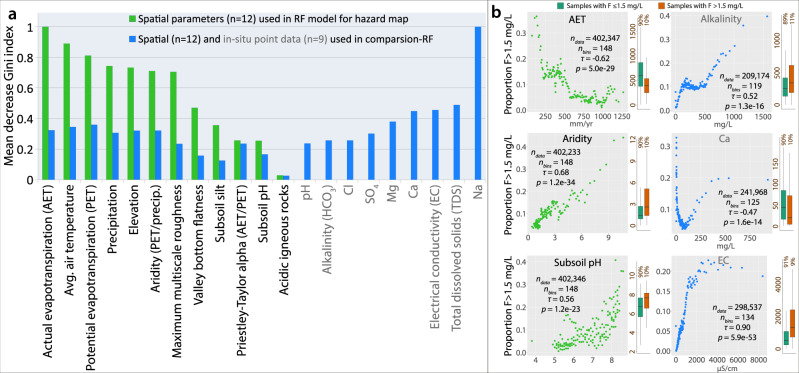
Environmental and hydrochemical conditions related to high fluoride concentrations in groundwater. **a** Importance of the predictor variables in modeling in terms of mean standardized decrease of the Gini index, which measures classification impurity such that a reduction represents improved classification. The importance of the 12 variables of the final (geospatial) model (Fig. 1) is shown in green. For comparison, the importance of the combined geospatial/in-situ groundwater variables in the mixed (non-geospatial) model is shown in blue, for which it is not possible to generate a prediction map. **b** Correlation between selected variables and the proportion of groundwater fluoride measurements greater than 1.5 mg/L as histograms (see “Methods”). The plots of actual evapotranspiration, aridity, and subsoil pH are taken from the model using only geospatial parameters. The number of data points, number of bins (points), Kendall rank correlation (*τ*), and associated *p*-value (*p*) are indicated. The vertical box plots indicate the distribution of each parameter associated with fluoride concentrations ≤1.5 mg/L and >1.5 mg/L, with the central line representing the median, the hinges showing the 25th and 75th percentiles, and the whiskers extending up to 1.5 times the inter-quartile range. For ease of presentation, outliers are not displayed.

A random forest model was also run incorporating chemical groundwater parameters measured in situ along with fluoride. Although this cannot be used to generate a map, it helps to illuminate the geochemical conditions that are favorable for the occurrence of high fluoride concentrations in groundwater. The importance of the predictor variables in the random forest model in terms of decrease in the Gini index indicates the relative strength of each variable (Fig. 2a). The green columns in the figure show the relative importance of the model using only spatially continuous variables, whereas the blue columns indicate the importance of the variables used in the random forest with a combination of both geospatial and in-situ groundwater parameters. The same geospatial variables were used along with nine in-situ chemical parameters with the greatest frequency in the dataset and highest importance as determined by RFE. The inclusion of in-situ groundwater parameters did improve the performance of the model, as demonstrated by a balanced accuracy of 0.89, a kappa of 0.77, and an AUC is 0.95 (versus 0.82, 0.64, and 0.90, respectively, for the spatially continuous model). Although the variable of acidic igneous rocks was added due to its documented association with fluoride concentrations in groundwater, the inclusion of further lithological categories had a negligible effect on the model results and were therefore not incorporated.

### Conditions affecting fluoride accumulation

While predictor importance is dominated by climate-related parameters, some of the in-situ groundwater parameters also point to climate-related signals, such as evaporative concentration of Na and EC in arid regions^7,28,29^ (Fig. 2a). To better assess these relationships with the large compiled dataset, plots were made of the proportion of high fluoride measurements against bins of each parameter (Supplementary Figs. 9 and 10). Selected key results are shown in Fig. 2b.

The climate variables (Fig. 2b) clearly indicate a strong correlation between high fluoride concentrations and a dry climate. This is, for example, illustrated by the strong positive linear relationship between high fluoride and aridity (potential evapotranspiration (PET)/precipitation). Conversely, actual evapotranspiration (AET) is negatively correlated with high fluoride due to greater AET implying a greater availability of water and hence more humid conditions. The relationship with a dry climate is consistent with the salinity of the groundwater samples, with chloride (Cl), electrical conductivity (EC), and total dissolved solids (TDS) as well as boron (B) and sodium (Na) showing strongly positive correlations with high fluoride. These all appear to plateau at very high levels, for example at 3000 µS/cm for electrical conductivity (Fig. 2b), where fluoride concentrations are likely controlled by sorption equilibria and saturation in water. Furthermore, both subsoil pH and groundwater pH show strong positive trends with high fluoride concentrations that steepen at higher pH levels. As with pH, the prevalence of high fluoride increases with alkalinity (HCO_3_)^7,46–48^, though with a distinct plateau between about 100–500 mg/L where the proportion of *F* > 1.5 mg/L stays near 0.1. This shows that low fluoride concentrations are maintained in some 90% of the cases, whereas alkalinity >500 mg/L is associated with increasing incidences of high fluoride waters (Fig. 2b).

High pH is consistent not only with aridity but also with hard water. At lower concentrations, the occurrence of high fluoride is strongly negatively correlated with calcium (Ca^2+^) and magnesium (Mg^2+^), which confirms previous results^7,9,14,49,50^. However, the trend then shifts abruptly and becomes strongly positive before showing no incremental effect at further increasing concentrations. For example, the prevalence of fluoride drops precipitously for calcium concentrations up to about 80 mg/L and then rises sharply again to about 400 mg/L, where it levels off at higher concentrations (Fig. 2b). Similar trends are observed with aluminum (Al), magnesium (Mg) and, to a lesser extent, barium (Ba) (Supplementary Fig. 9). The increase of fluoride with high levels of Ca and Mg is likely caused by evaporative conditions and increasing salinity^7^ as expressed by several related parameters, for example, high EC, TDS, Na, and Cl. Strong positive relationships are also found between high fluoride and bromide (Br), lithium (Li), molybdenum (Mo), potassium (K), sulfate (SO_4_), strontium (Sr), and uranium (U) (Supplementary Fig. 9). Finally, groundwater with higher temperatures is observed to contain higher fluoride concentrations, which is likely due to fluoride association with geothermal waters^9,42^.

### Population at risk of exposure

**Fig. 3:**
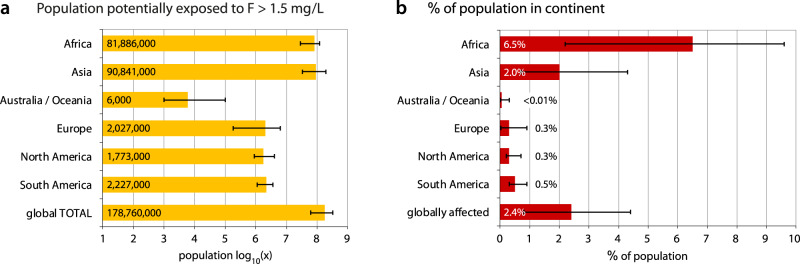
Population potentially exposed to fluoride >1.5 mg/L in groundwater. Calculations are made according to the hybrid approach, with **a** the number of people potentially affected by continent and **b** the percentage of the total population that this represents. The error bars represent the low and high estimates of affected population (see text).

The population consuming groundwater that is potentially exposed to fluoride was calculated by combining the predictions of the fluoride hazard model (Fig. 1) with the best available estimates for each country for the domestic use of unfiltered groundwater in both rural and urban settings^51^. After taking groundwater usage into account with global population data from 2020^52^, a range of population counts was calculated (Fig. 3) by multiplying the population everywhere by the probability of high fluoride (Supplementary Table 3, high estimate), as well as by counting the population only in those cells exceeding a probability cutoff of 0.5 but not multiplying by the probability (Supplementary Table 3, low estimate). A hybrid, intermediate approach of multiplying the population in cells by the modeled probability for cells with a probability greater than 25% was also carried out (see Supplementary Table 3 and Methods for details). The calculated total population at risk of exposure to fluoride in drinking water at concentrations greater than 1.5 mg/L is in the range of 63–330 million people, with 179 million people being estimated by the hybrid approach (Fig. 3a). This corresponds to 0.8–4.4% of the global population (2.4% with hybrid approach), with Africa being most heavily impacted with 2.2–9.6% of the population (6.5% with hybrid approach) (Fig. 3b).

## Discussion

The geospatial prediction model is dominated by climate parameters. In the combined model, these same variables are comparably important to the in-situ chemical parameters, which themselves also have a climate signature (related to aridity). This is not surprising given the relative abundance of fluorine in Earth’s crust and that climatic conditions control the water cycle, relate directly to the evaporative concentration of fluoride, and affect soil pH, and thereby fluoride retention. In addition, climate can be influenced by topography (e.g., orographic controls on cloud formation and wind patterns), which is also represented in the model through three topographic variables.

Despite geochemistry playing an integral part in the dissolution of fluoride in groundwater, only one geochemistry variable (acidic igneous rocks) was included in the final geospatial model, along with the two indirectly related variables of subsoil pH and silt. The muted influence of lithology on the model can be ascribed to the relatively low degree of detail in the available standardized global lithological map. If creating a prediction map at a smaller scale, the incorporation of country-scale lithological maps can indeed play an important role^43,53^. It is also worth noting that with regard to the calculation of variable importance, a categorical variable (such as lithology) is less likely than a continuous variable to have an effect on the model when its values are randomly sorted, due to having far fewer possible values. Although the inclusion of in-situ groundwater parameters in the combined model improved the model’s accuracy, they cannot be used in creating a prediction map due to these measurements representing data points rather than spatially continuous variables. However, if a sufficient density of measured hydrochemical parameters (e.g., Na, TDS, and EC) would exist in a given area, it could be possible to create gridded maps of these parameters to then use in statistical modeling and the creation of a prediction map of the area. This could be expected to improve the model’s accuracy, as with the non-geospatial model described above, though the extent of the prediction map would necessarily be limited by that of the gridded in-situ parameters. For this reason, it is not possible to create a global prediction map of fluoride based on gridded in-situ parameters.

Despite generalizing across the entire globe, the geospatial model (Fig. 1) is largely consistent with previous studies that created random forest models focused on India^38^, Ghana^53^ and parts of the American Southwest^40^ and similar, though less detailed, to another fluoride prediction model created for China^54^. In addition to areas well constrained by fluoride measurements, such as much of Australia and the Americas, it is noteworthy that the model identifies many regions with high fluoride hazard where no measurements were available, for example, in many parts of Africa and Central Asia. This is possible due to similar environmental conditions to those where many fluoride samples exist, such as North Africa (few measurements) versus central Australia (many measurements). Indeed, this highlights the utility of a prediction model in identifying areas of concern where more groundwater testing should be conducted to mitigate human health risks.

In spite of an uneven geographical distribution of fluoride data points, the model predictions and performance are reasonably similar across all continents (Supplementary Table 5). For example, the mean model probabilities by continent range between 0.11 (North America) and 0.32 (Africa) and do not appear related to the proportion of high measurements. The mean AUC calculated separately with the test data points from each continent ranges between 0.83 and 0.91, with the low end of this range (Europe) still being on par with or better than results from comparable country-wide studies^38,43,55,56^. Although the highest AUC and balanced accuracy are found in the continents containing the largest proportion of the dataset (i.e., North America with 60%, Australia with 15%, and Africa with 12%), the next-best performing continent is South America, which happens to contribute the least amount of data (1%) to the dataset.

Due to varying patterns of population density and groundwater usage, the implications of groundwater fluoride hazard (Fig. 1) for human health risk differ considerably. The hybrid approach to calculating the affected population produces the estimate of 179 million people (2.4% of the global population) and, as such, lies between the counts of the other two methods, which can be considered end-member extremes that are most probably under or overestimates. Furthermore, with 179 million being congruent with the previously published estimate of 200 million^1^ and the hybrid approach itself making intuitive sense (see “Methods”), we consider the hybrid calculations to offer the best estimates of affected populations, albeit with the broad ranges of population counts offered by the other two approaches.

Regardless of the considerable ranges of affected populations, the relative distribution remains fairly constant. Nearly all of the affected population resides either in Asia (51–59% of total) or Africa (37–46% of total), with all other continents in all cases making up about 1% or less (Fig. 3a and Supplementary Table 3). Indeed, among the top 20 countries in terms of at-risk population (hybrid approach), 14 are found in Africa and six in Asia (Table 1), with both of these continents being the most strongly affected with ~6% of the African population and ~2% of the Asian population potentially exposed to high fluoride concentrations in drinking water (Fig. 3b).

**Table 1:** Top 20 countries in population potentially affected by fluoride concentrations in groundwater greater than 1.5 mg/L.

Rank	Country	Population at risk (range)	Rank	Country	Population at risk (range)
		(million)			(million)
1	India	49 (26–89)	11	Malawi	4.0 (3.5–4.8)
2	China	22 (1–50)	12	Zambia	3.4 (1.4–3.6)
3	Dem. Rep. Congo	15 (2–16)	13	Mozambique	2.6 (1.7–3.4)
4	Ethiopia	9.6 (4.0–13.8)	14	Angola	2.2 (0.7–2.4)
5	Pakistan	7.6 (2.3–14.5)	15	Afghanistan	1.7 (0.5–4.8)
6	Kenya	7.5 (4.2–8.3)	16	Cameroon	1.6 (0.3–2.5)
7	Nigeria	7.4 (1–17)	17	Madagascar	1.4 (0.7–2.3)
8	Tanzania	6.9 (3.7–7.9)	18	Chad	1.2 (0.1–2.2)
9	Uganda	4.8 (0.9–8)	19	Niger	1.2 (0.2–2.6)
10	Yemen	4.3 (2.6–4.4)	20	Myanmar	1.1 (0.07–3.3)

The first number was calculated by multiplying the groundwater-consuming population of each cell by the probability of groundwater exceeding 1.5 mg/L for cells with a probability >0.25 (hybrid approach). The range in parentheses is produced by the approaches of taking the full groundwater-consuming population of cells with a probability >0.50 (low estimate), as well as applying the probability to all map cells (high estimate).

**Fig. 4:**
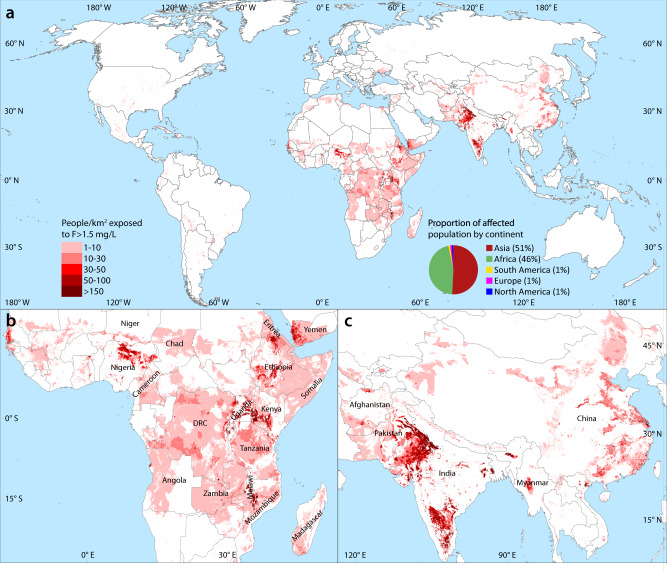
Estimated population potentially exposed to fluoride concentrations in drinking water greater than 1.5 mg/L. **a** Global map and break-down by continent. Detailed views of **b** sub-Saharan Africa and southern Arabian peninsula and **c** south and east Asia with the most strongly affected countries indicated. The population was calculated with the hybrid approach (see text) for areas with a greater than 25% probability of incurring high fluoride in groundwater (Fig. 1) by multiplying the total population by the hazard percentage and the proportion of domestic water usage coming from untreated groundwater^51^.

Two regions with large potentially affected populations for which only relatively few direct measurements of groundwater quality were available to constrain the model are China and Central Africa (Fig. 1b and Supplementary Fig. 1). The model also indicates a particularly elevated fluoride risk across much or most of Angola, Cameroon, Chad, Democratic Republic of the Congo (DRC), Ethiopia, Eritrea, Kenya, Madagascar, Malawi, Mozambique, Nigeria, Somalia, Tanzania, Zambia, and Zimbabwe as well as Yemen (Table 1). The at-risk population figures provide only a rough estimate of the actual number of people affected, which can only be verified by epidemiological studies on the ground. Nevertheless, Fig. 4 provides a meaningful broad-scale indication of where such investigations are most needed.

In warmer regions, a lower limit of fluoride in drinking water (e.g., 1.0 mg/L) may be advisable due to increased water consumption needs^57^, which suggests that the health risk in affected tropical and sub-tropical zones (e.g., Central Africa or South Asia) may be even greater. As such, future temperature increases would likely exacerbate the situation. Furthermore, where climate change leads to increased aridity, fluoride concentrations in groundwater could experience a long-term increase (assuming the adequate presence of fluoride-bearing minerals), as indicated above with aridity being closely linked to high fluoride concentrations and associated increases in pH, alkalinity, and aquifer residence times. This effect could also lead to an increased reliance on groundwater to compensate for less reliable surface-water supplies.

The key solutions to coping with these present and future challenges include testing wells and springs in fluoride-prone areas and implementing corrective measures, where necessary. Solutions could include, for example, switching/blending water sources or engaging various defluoridation methods^3,20,27,34,58^. The hazard and risk maps presented above offer an important first step on this path.

## Methods

### Fluoride dataset and additional water parameters measured in situ

Geo-located fluoride measurements from 402,452 unique wells and springs from 77 countries were compiled from 80 published or otherwise publicly available sources (Supplementary Table 1). If more than one measurement was available from a given well/spring, the average of the measurements was taken. Well depth and 41 other measured physicochemical water parameters, where reported, were also assembled (Supplementary Fig. 9 and Supplementary Table 6).

These measurements (along with spatially continuous parameters used in modeling mentioned below) were analyzed with respect to their associated fluoride concentrations meeting or exceeding the WHO guideline concentration of fluoride in drinking water of 1.5 mg/L in order to identify possible relationships with the occurrence of high fluoride. Each parameter was first ordered and placed into histograms of equally sized bins (of variable width), for each of which the prevalence of high fluoride measurements (>1.5 mg/L) was calculated. The number of bins used with each parameter was determined according to the Rice rule, which is twice the cubed root of the number of observations ($\root 3 \of {n}\times 2$). For the number of data dealt with here, this generally results in a much larger number of bins than that determined according to the more commonly used Sturges’ rule (${{{{{{\rm{log }}}}}}}_{2}(n)+1$) and thereby potentially avoids problems of over-smoothing^59^. Kendall rank correlations were then calculated between the prevalence of high fluoride concentrations and the median parameter value in each bin. This correlation type is not sensitive to the distribution, which for the data at hand is often skewed rather than normal. Boxplots were also created for each parameter in the two categories of low (≤1.5 mg/L) and high (>1.5 mg/L) fluoride.

### Spatially continuous parameters

Initially, 62 spatially continuous environmental parameters that may relate either directly or indirectly to the processes of the geogenic accumulation of fluoride in groundwater^38^ were assembled as potential predictor variables (Supplementary Table 2). These generally fall into the categories of climate, geology, soil, or topography. Any broad anthropogenic impacts related to agriculture or urbanization could be considered through land use. The resolution of the spatial datasets ranges between 7.5” (~250 m) and 30” (~1 km). A subset of these parameters was later selected for modeling (see *Random forest modeling* below). Although most of these parameters are available as continuous values, several categorical datasets of geology, land use, and soil classification were also considered. A primary goal in conducting machine learning modeling here is to create a prediction map of high fluoride concentrations in groundwater. As such, only spatially continuous data, i.e., not the 42 in-situ point data mentioned in the previous section, could be used as predictor variables for this purpose. The values of the predictor variables were taken according to the geographic coordinates of the fluoride concentrations.

### Random forest modeling

Among the various available machine-learning algorithms, the random forest method^41^ was chosen due to our experience in its ability to efficiently produce highly accurate models^37,38,44^. The computational efficiency of random forest is also particularly relevant here due to the large number of data being utilized. Classification modeling was conducted in order to focus on the health risk of consuming drinking water with fluoride concentrations greater than 1.5 mg/L. Therefore, the fluoride data were converted into binary format based on meeting or exceeding this guideline. Furthermore, by not modeling continuous values, the unknown and potentially highly variable measurement errors from the great diversity of data sources, as well as any temporal changes in concentrations, become irrelevant when the measurements are clearly greater or less than 1.5 mg/L.

A random forest averages together many decision trees to form a single composite model. Randomness is introduced in the growing of each tree by bootstrap aggregating, also known as bagging or sampling with replacement, the data rows with which to form a tree as well as by considering only a limited number of randomly selected predictor variables, in this case, the square root of the total number of predictors, at each node. The modeling was implemented with the R programming language^60^ and the ranger package^61^.

In order to identify particularly relevant parameters as well as reduce model complexity, the most important predictor variables were identified through recursive feature elimination with the varSelRF package^62^, whereby the 20% least important variables are removed in successive iterations. The final selected combination is that with the smallest number of features and an error rate that is within one standard deviation of the minimum error rate of all forests. Once the final predictor variables were selected, the minimum number of samples to require at a node were then tuned using the caret package^63^ (values from 1 to 5).

Model performance was evaluated through 100-fold cross validation using an 80%-training/20%-testing split, which was stratified by the prevalence of high fluoride. That is, the proportion of high fluoride cases of 0.102 was always maintained in the randomly selected training and testing datasets. Each random forest was grown with 1001 trees. (Doubling the iterations made no improvement in accuracy.) Bagging was made preferentially from high fluoride cases at a rate of 1-prevalence (0.898) such that each tree was grown with equal numbers of both low and high fluoride classes. The prediction results from the 100 cross validations were averaged. Measures of performance included sensitivity (proportion of high fluoride cases correctly classified), specificity (proportion of low fluoride cases correctly classified), balanced accuracy (average of sensitivity and specificity), area under the ROC (receiver operator characteristic) curve (AUC), which considers combined sensitivity and specificity over all probability cutoffs and ranges from 0.5 to 1 and kappa, which adjusts the accuracy (acc) of the model by the no information rate (NIR; proportion of majority class) (Eq. 1):

1 $$\kappa \equiv \frac{{{{{{\rm{acc}}}}}}-{{{{{\rm{NIR}}}}}}}{1-{{{{{\rm{NIR}}}}}}}$$

In order to improve the interpretability of kappa, the testing dataset was balanced by randomly down-sampling the majority class (low fluoride) to equal the size of the minority class (high fluoride) before the confusion matrix was calculated, which includes kappa, sensitivity, and specificity. This was repeated ten times and the results averaged for the cross validation of each random forest.

The final random forest model was grown with the full dataset. The effect of the predictor variables on the model was evaluated through importance as measured in the random forest and Kendall rank correlations between binned values of each predictor and the prevalence of high fluoride, as described above under *Fluoride dataset and additional water parameters measured* in situ. Importance was determined as the mean decrease of the Gini index (classification impurity), corrected for bias in number of categories, when the values of a variable are randomly re-sorted. A global probability map of the occurrence of fluoride concentrations greater than 1.5 mg/L was then created by applying the final model to the predictor datasets. Predictors with 1 km resolution were first reformatted to a 250  pixel size, which did not affect their values as none were based on an area measurement.

### Population risk estimation

A population risk map was developed by applying the hazard probability map to global rural and urban population estimates for 2020^52^ along with the latest available country-level rural and urban use rates of untreated groundwater^51^. Two fundamentally different approaches were considered in running the calculation. In the first approach, the population was multiplied by the probability of high fluoride. For example, for a 250 m × 250 m cell with a model prediction of 0.47, 47% of the population in that cell would be counted. Despite counting fewer people in direct accordance with the modeled probability, densely populated areas with non-trivial groundwater usage would still add considerably to the overall count despite only a minimal chance of having high fluoride concentrations in groundwater. In the other approach, a probability cutoff is used to determine the areas with a sufficiently high modeled probability and take only those cells into account. A reasonable cutoff to use is the probability at which the sensitivity (true positive rate) and specificity (true negative rate) of the model in predicting the test data are equal. This was approximately 0.5, which reflects the balanced training data that were used in growing each random forest. While this method accounts for only the best-determined cells, it neglects those cells that fall just below the probability threshold and may still be at least somewhat affected by high fluoride concentrations.

In order to take advantage of the benefits of both approaches while avoiding their shortcomings, a hybrid approach was also taken by considering only areas with a probability of half of the sensitivity-specificity crossover (0.25) and multiplying the groundwater-consuming populations in these areas by the model probabilities. Compared to the first approach of multiplying the population of each cell by its probability, areas with a very low probability of high fluoride (≤0.25) are not counted. With respect to the cutoff-at-0.5 approach, the hybrid approach accounts for areas somewhat below the optimal cutoff while applying a decreasing weight as the probability decreases to 0.25, as well as, of course, an increasing weight for higher probability cells. As such, this method seems to be the most reasonable, realistic, and unbiased.

## Supplementary information

Supplementary Information

Description of Additional Supplementary Files

Supplementary Data 1

## Data Availability

The global fluoride prediction map and population risk map (Figs. 1, 4) have been deposited as GeoTIFF rasters in the ERIC/open database^64^ (10.25678/0006GQ) and can also be viewed on the Groundwater Assessment Platform (www.gapmaps.org). The raw data used to generate these maps are in general protected and are not available due to data privacy laws. Interested readers are instead referred to the data sources listed in Supplementary Tables 1 and 2.

## Code availability

The code used in generating this paper’s results along with a sample dataset are available at the ERIC/open database^64^ (10.25678/0006GQ).
