# Supplementary material for: Global analysis and prediction of fluoride in groundwater
Source: Nat Commun. 2022 Aug 1;13:4232. doi: 10.1038/s41467-022-31940-x (PMC9343638; doi:10.1038/s41467-022-31940-x)
Supplement: Supplementary file 1 — Supplementary Information [file 41467_2022_31940_MOESM1_ESM.pdf]

Supplementary Information for:

Global analysis and prediction of fluoride in groundwater

Joel Podgorski[1] and Michael Berg[1]

[1]Department of Water Resources and Drinking Water, Eawag, Swiss Federal Institute of Aquatic Science and Technology, 8600 Dübendorf, Switzerland

**Corresponding authors. Email: joel.podgorski@eawag.ch, michael.berg@eawag.ch*

Supplementary Figure 1: Fluoride data points

Supplementary Figure 2: Frequency of fluoride concentrations

Supplementary Figure 3: Global groundwater fluoride prediction model, focused on North America

Supplementary Figure 4: Global groundwater fluoride prediction model, focused on Europe

Supplementary Figure 5: Global groundwater fluoride prediction model, focused on Asia

Supplementary Figure 6: Global groundwater fluoride prediction model, focused on South America

Supplementary Figure 7: Global groundwater fluoride prediction model, focused on Africa

Supplementary Figure 8: Global groundwater fluoride prediction model, focused on Australia and neighboring Oceania

Supplementary Figure 9: Correlations between in-situ parameters and proportion of groundwater fluoride measurements greater than 1.5 mg/L

Supplementary Figure 10: Correlations between continuous geospatial parameters and proportion of groundwater fluoride measurements greater than 1.5 mg/L

Supplementary Table 1: Summary and sources of groundwater fluoride measurements

Supplementary Table 2: Spatially continuous environmental parameters considered in analysis and modeling

Supplementary Table 3: Fluoride hazard areas and at-risk groundwater-consuming populations by continent

Supplementary Table 4: Summarized statistics of 100 cross validations of random forests

Supplementary Table 5: Comparison of model predictions and performance of the global model across all continents

Supplementary Table 6: Descriptive statistics of other parameters measured in situ

References in Supplementary Materials

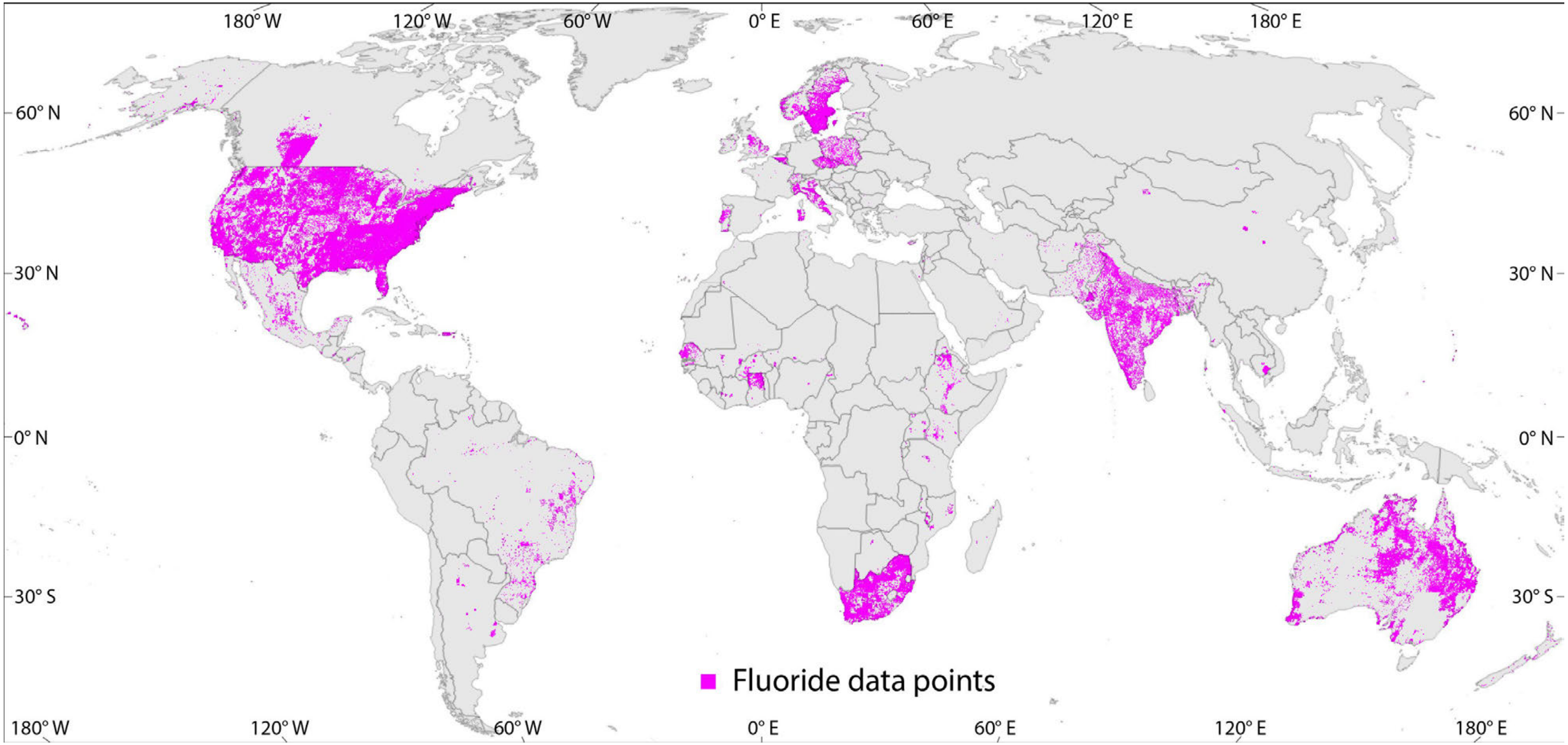

Supplementary Figure 1: Fluoride data points (n= 402,452) used in analysis and modeling. The data sources are listed in Supplementary Table 1.

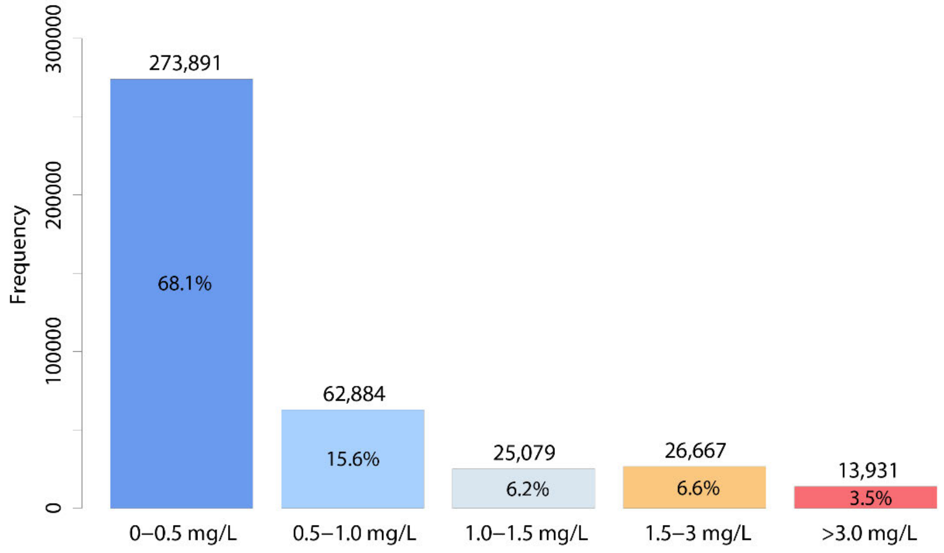

Supplementary Figure 2: Frequency of concentration ranges of the fluoride data points (n= 402,452) used in analysis and modeling. The data sources are listed in Supplementary Table 1

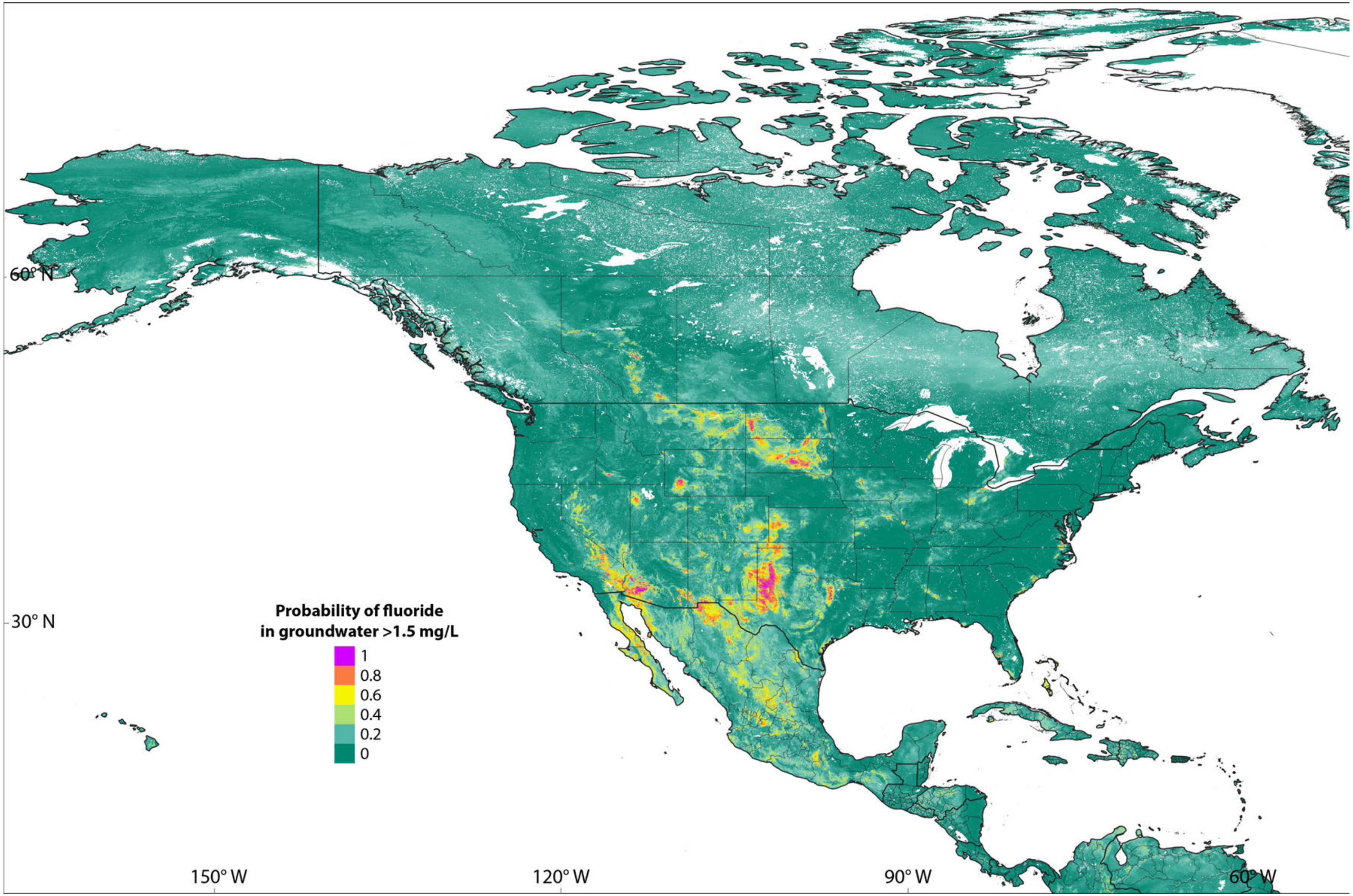

Supplementary Figure 3: Global groundwater fluoride prediction model (as shown in Fig. 1), focused on North America.

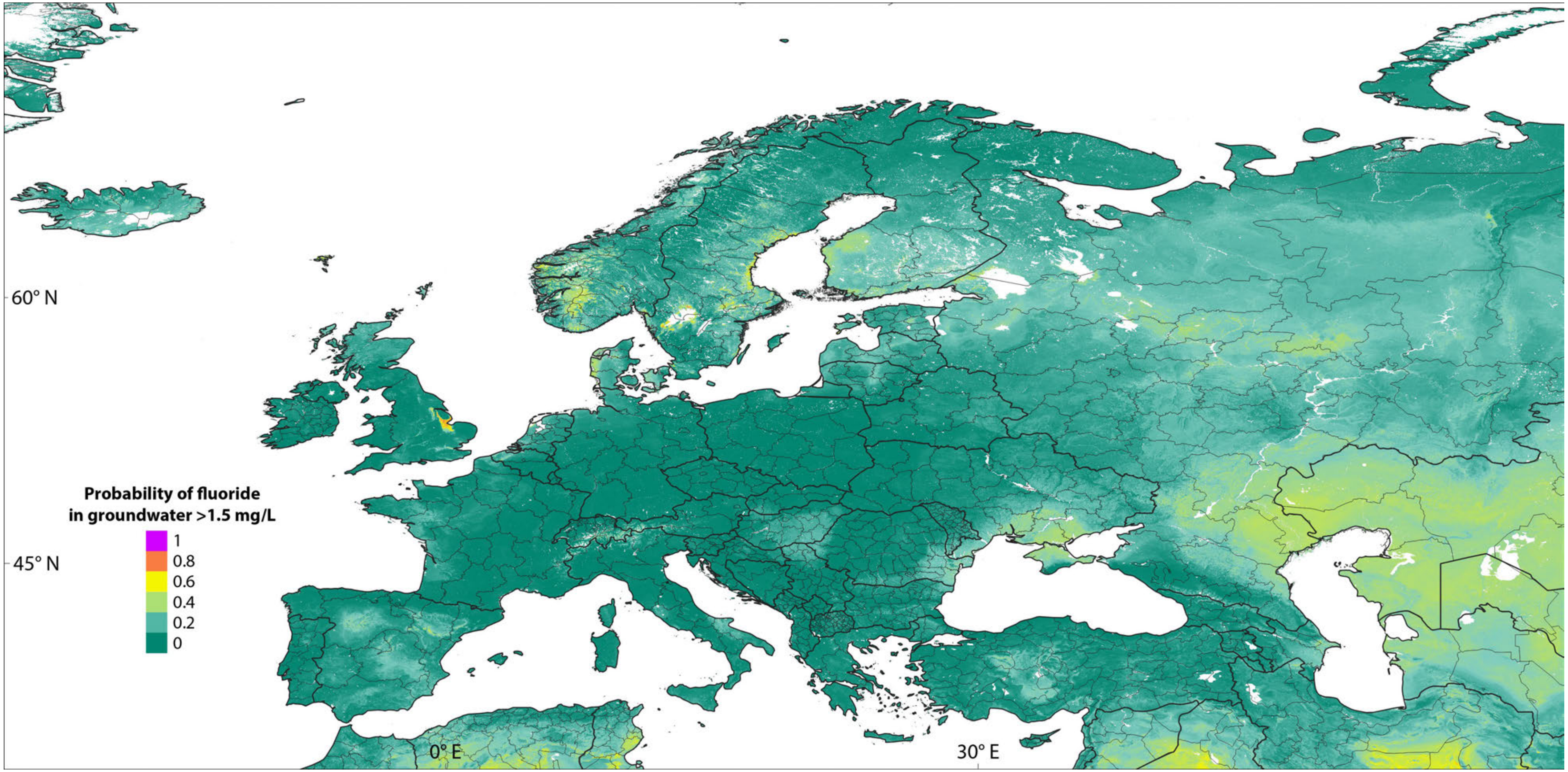

Supplementary Figure 4: Global groundwater fluoride prediction model (as shown in Fig. 1), focused on Europe.

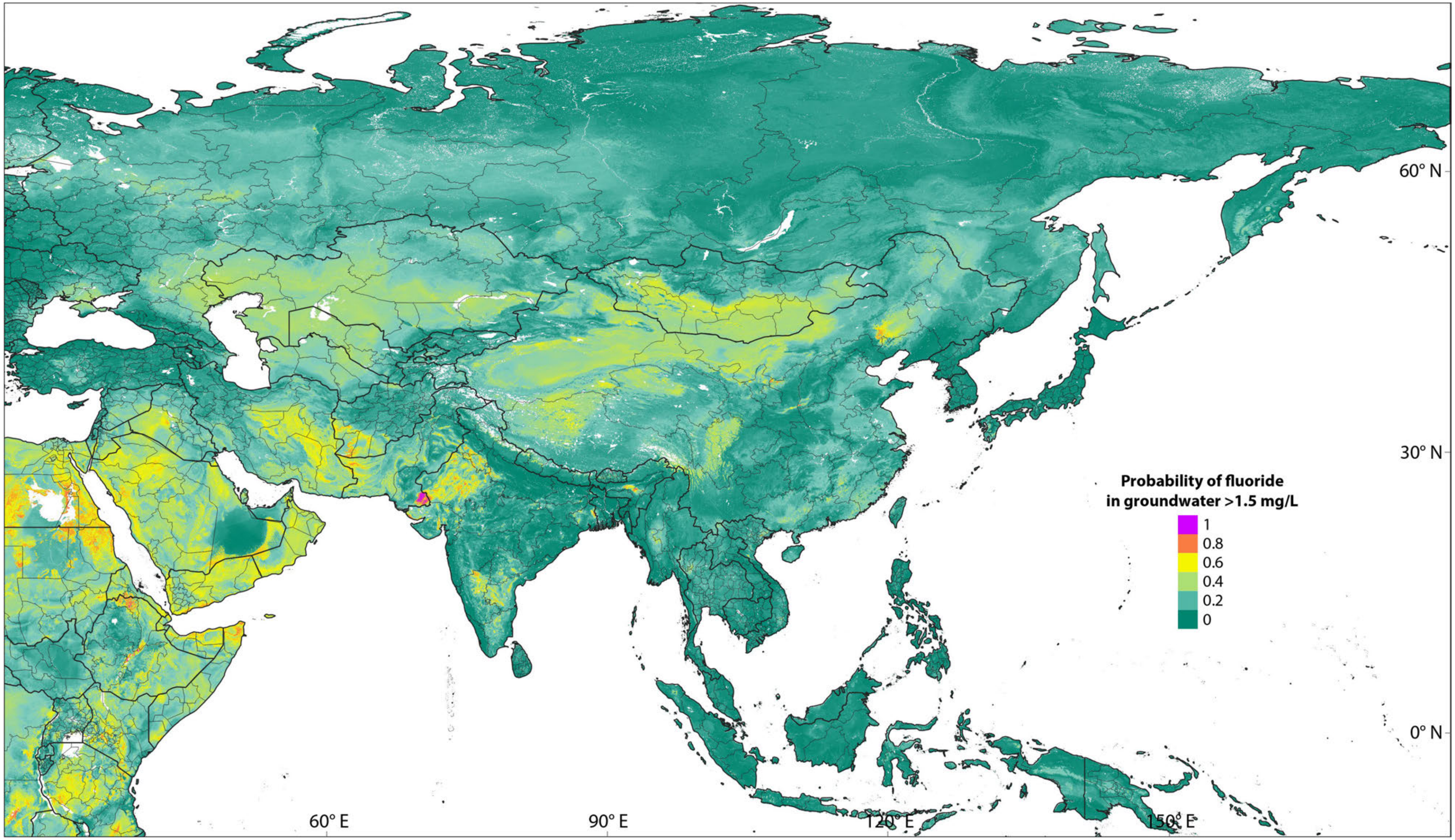

Supplementary Figure 5: Global groundwater fluoride prediction model (as shown in Fig. 1), focused on Asia.

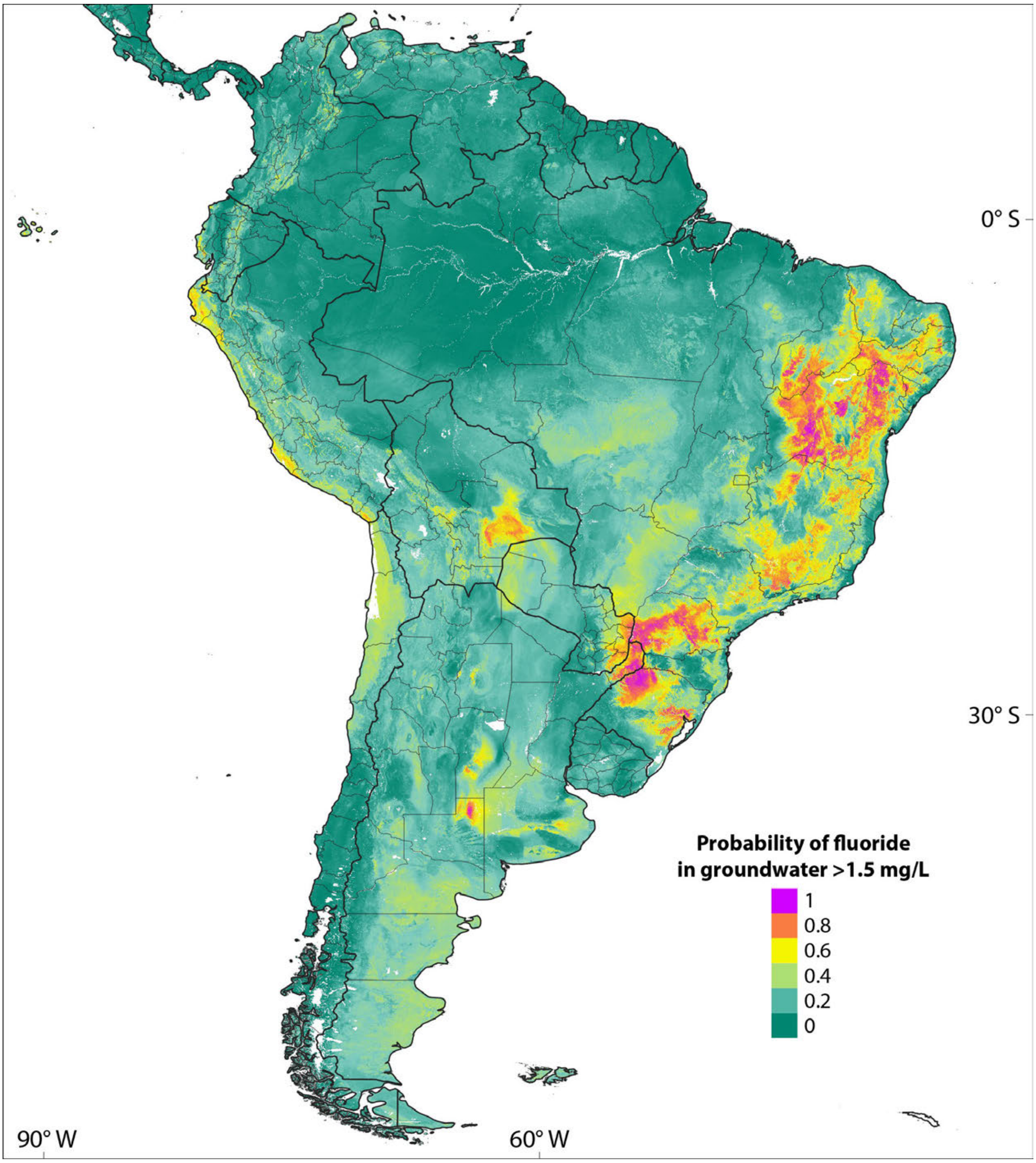

Supplementary Figure 6: Global groundwater fluoride prediction model (as shown in Fig. 1), focused on South America.

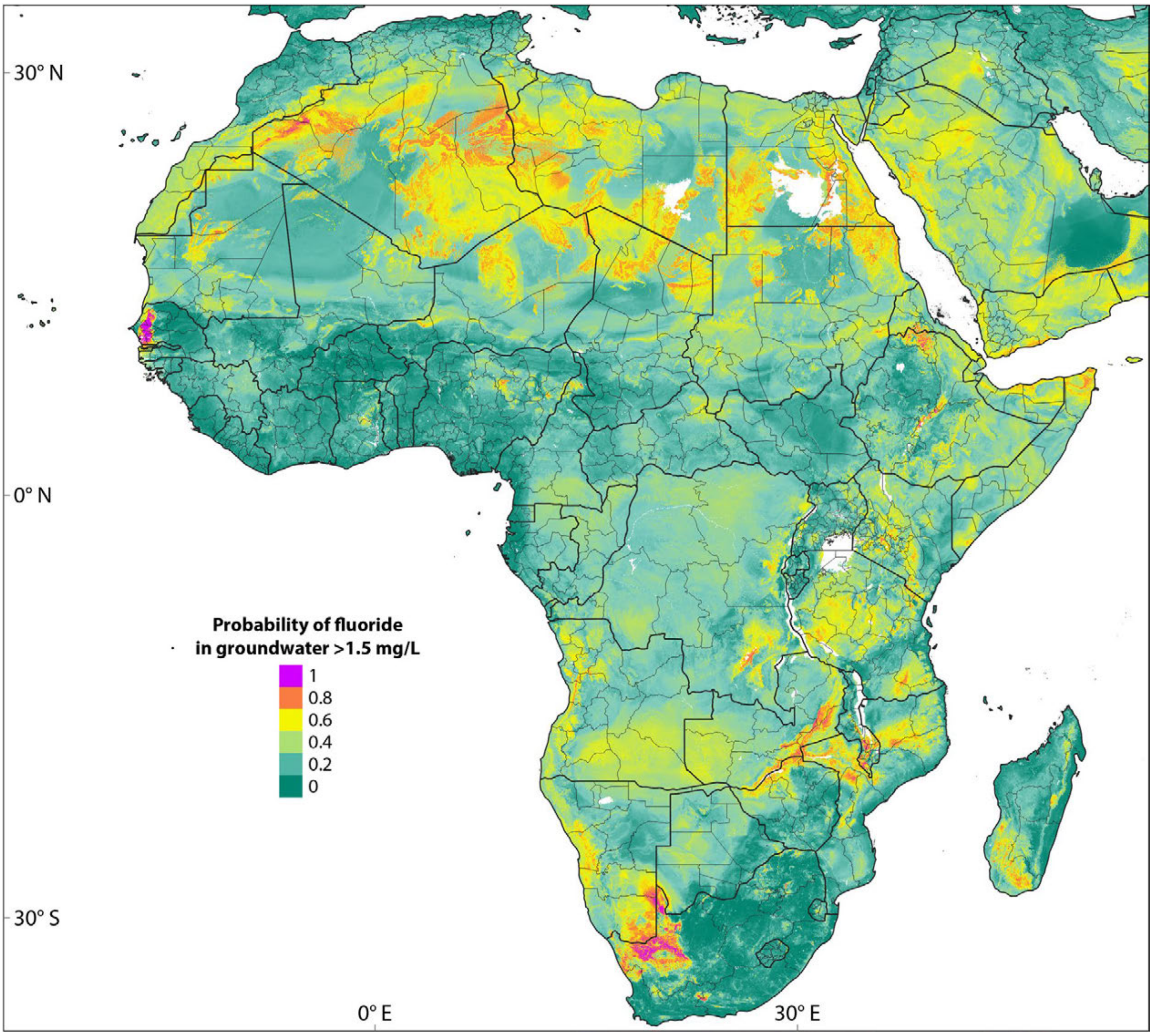

Supplementary Figure 7: Global groundwater fluoride prediction model (as shown in Fig. 1), focused on Africa.

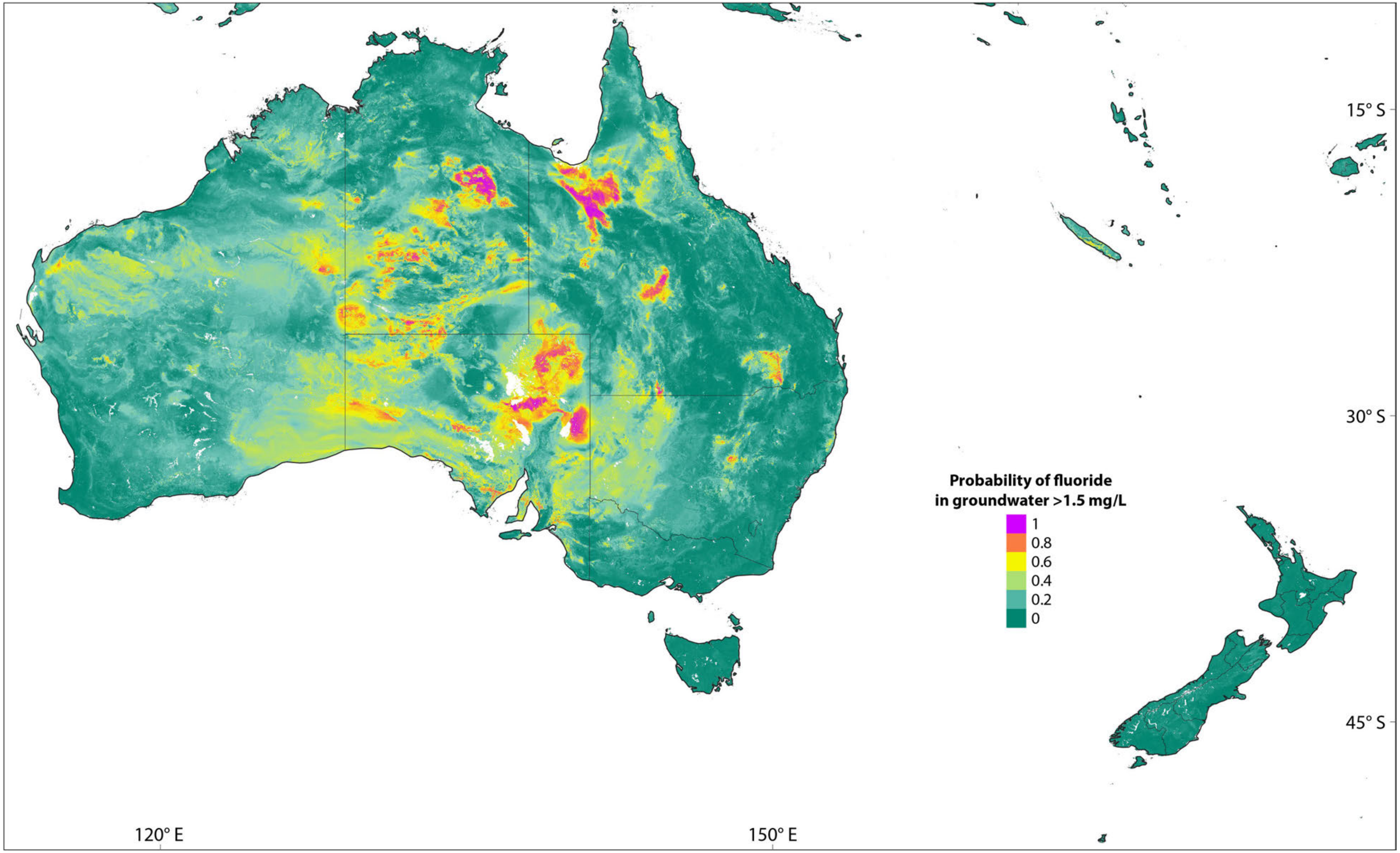

Supplementary Figure 8: Global groundwater fluoride prediction model (as shown in Fig. 1), focused on Australia and neighboring Oceania.

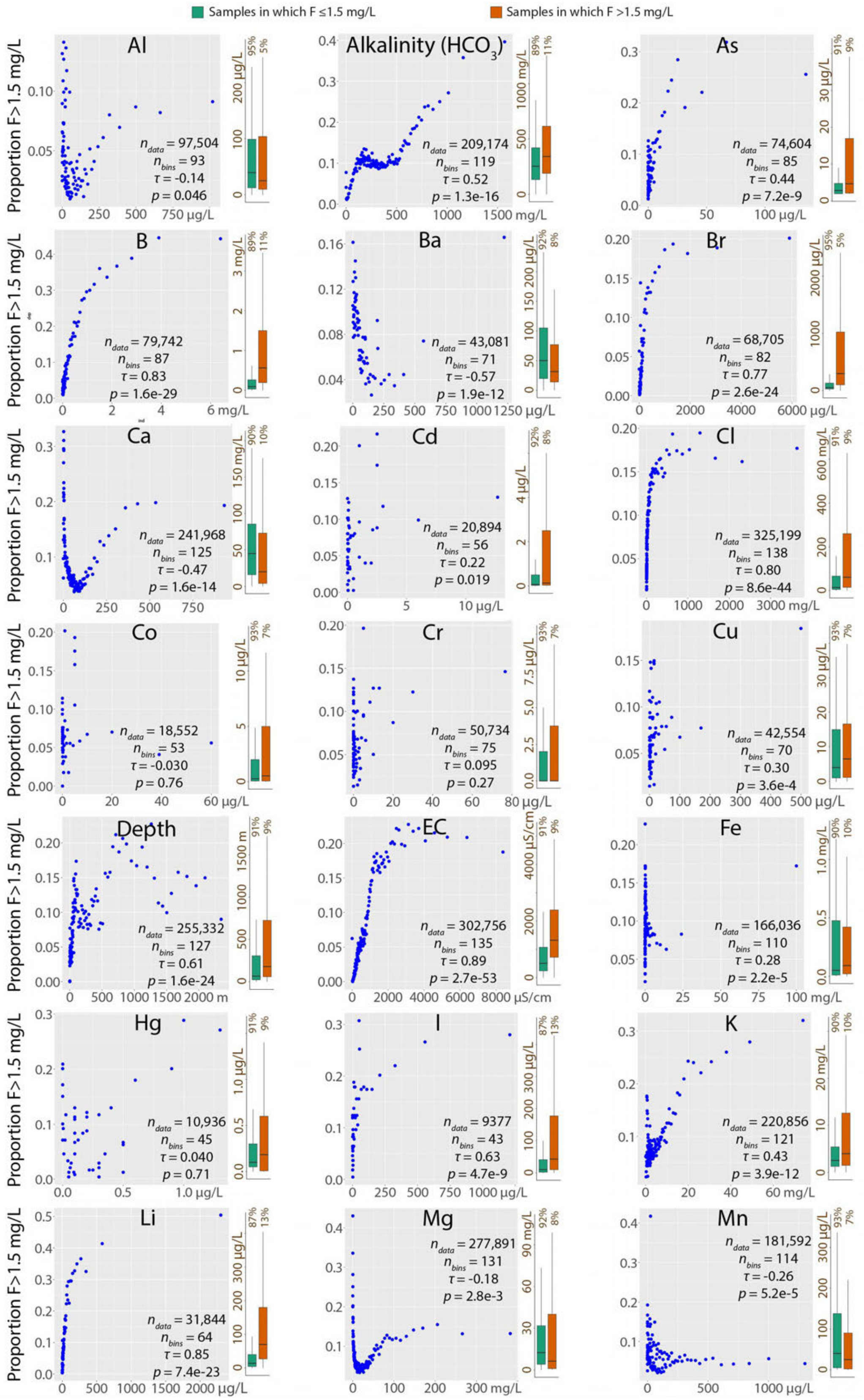

Supplementary Figure 9: Correlations between parameters measured in situ and the proportion of groundwater fluoride measurements greater than 1.5 mg/L. The number of data points, number of bins (points), Kendall rank correlation (τ) and associated p-value (*p*) are indicated (see text for explanation). The vertical box plots indicate the distribution of each parameter associated with fluoride concentrations ≤1.5 mg/L and >1.5 mg/L, with the central line representing the median, the hinges showing the 25th and 75th percentiles and the whiskers extending up to 1.5 times the inter-quartile range. For ease of presentation, outliers are not displayed.

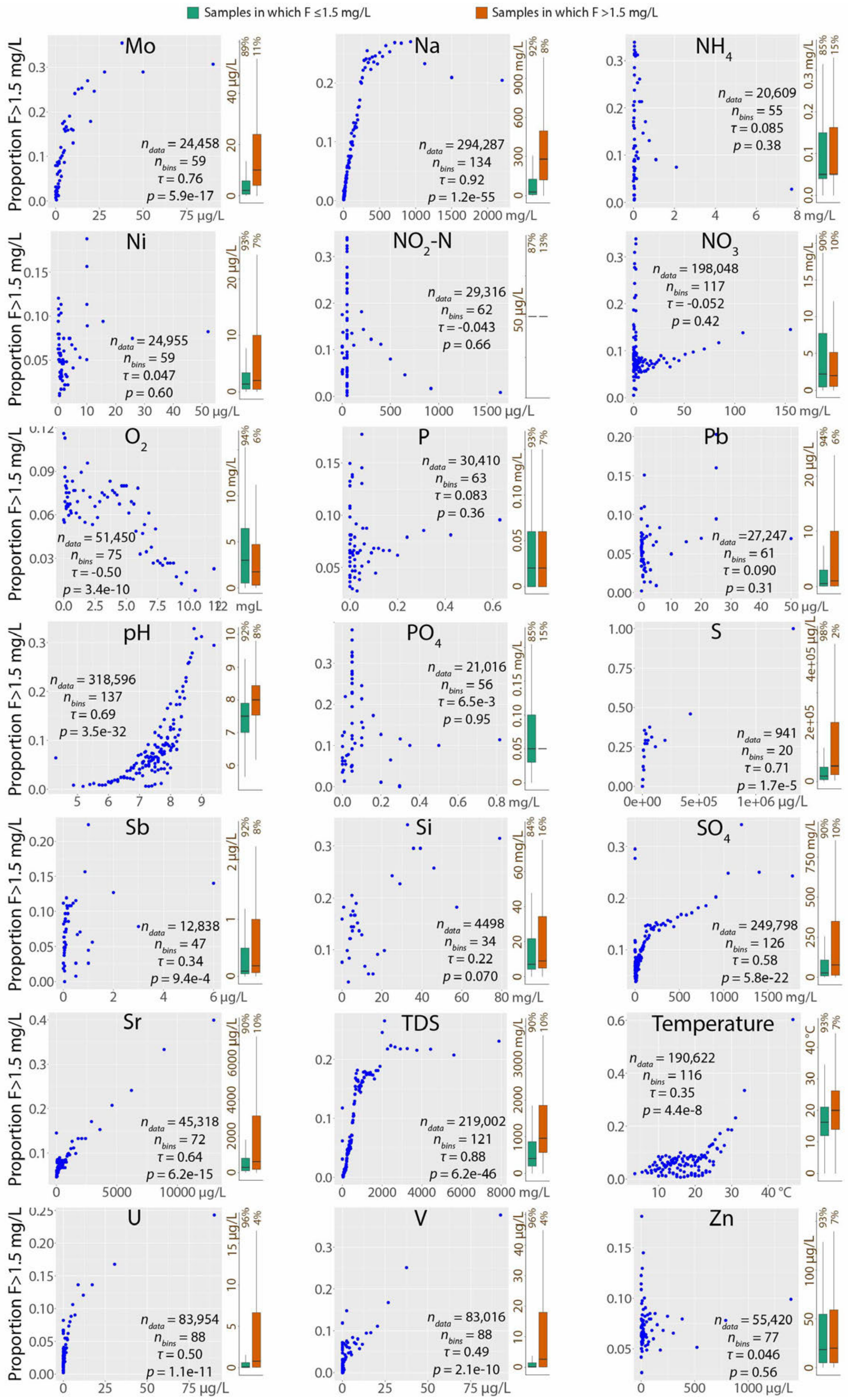

Supplementary Figure 9 (cont.)

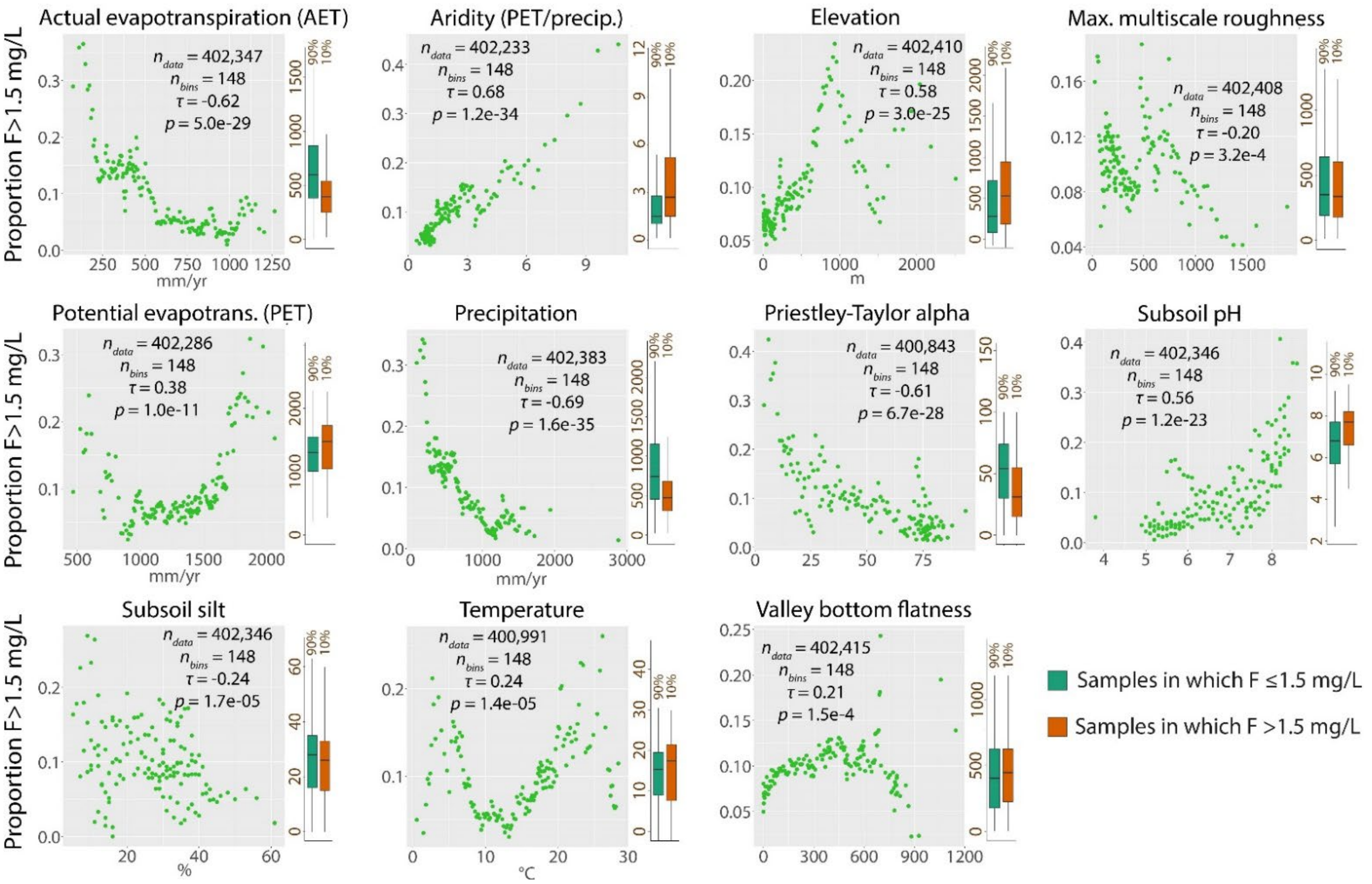

Supplementary Figure 10: Correlations between the continuous geospatial parameters used in the final geospatial model (Fig. 1) and the proportion of groundwater fluoride measurements greater than 1.5 mg/L. The number of data points, number of bins (points), Kendall rank correlation (τ) and associated p-value (*p*) are indicated (see text for explanation). The vertical box plots indicate the distribution of each parameter associated with fluoride concentrations ≤1.5 mg/L and >1.5 mg/L, with the central line representing the median, the hinges showing the 25th and 75th percentiles and the whiskers extending up to 1.5 times the inter-quartile range. For ease of presentation, outliers are not displayed.

Supplementary Table 1: Summary by country of the distribution of groundwater fluoride concentrations and sources used for analysis and modeling. IQR stands for interquartile range. In the case of a time series of measurements at a given well/spring, the average of the measurements was taken.

Country	Num. F data points	Range (mg/L)	Mean F (mg/L)	Median F (mg/L)	IQR F (mg/L)	Reference
Afghanistan	360	0-8.1	0.45	0.26	0.18-0.48	[1-3]
Algeria	10	0.51-2.45	1.78	1.82	1.70-2.17	[4]
Argentina	591	0-28.4	1.19	0.60	0.00-1.22	[3,5-10]
Australia	58,571	0-6487.4	1.15	0.30	0.11-0.70	[11-19]
Bangladesh	469	0.001-1.28	0.25	0.21	0.14-0.33	[3,20,21]
Belgium	309	0.035-3.50	0.15	0.11	0.07-0.16	[22]
Botswana	76	0-3.2	0.61	0.38	0.19-0.76	[23]
Brazil	2675	0.25-17.6	1.36	1.02	1.00-1.60	[10,24,25]
Bulgaria	2	0.53-0.58	0.56	0.56	0.53-0.58	[22]
Burkina Faso	273	0.046-2.55	0.28	0.19	0.11-0.31	[4,26]
Cambodia	7007	0-110	0.84	0.62	0.37-0.95	[27]
Cameroon	39	0.19-15.20	6.55	8.17	0.95-9.50	[28]
Canada	25,469	0-13.1	0.69	0.30	0.20-0.90	[29-33]
Chile	1	0.11-0.11	0.11	0.11	0.11-0.11	[3]
China	293	0.01-13.3	1.78	1.04	0.34-2.40	[34-38]
Cote d'Ivoire	102	0-4	0.40	0.20	0.10-0.30	[39]
Croatia	4	0.076-0.10	0.09	0.08	0.08-0.10	[22]
Cyprus	18	1.3e-4-3.3e-3	0.00	0.00	0.00-0.00	[22]
Czech Republic	651	0-0.006	0.00	0.00	0.00-0.00	[22]
Democratic Republic of the Congo	8	0.009-0.97	0.49	0.48	0.34-0.67	[4]
Denmark	21	1.5e-4-5.9e-4	0.00	0.00	0.00-0.00	[22]
Egypt	19	0.79-2.004	1.32	1.30	1.10-1.46	[40]
Eritrea	55	0.12-3.73	0.98	0.72	0.34-1.21	[41,42]
Estonia	33	0.14-3.31	0.80	0.63	0.30-1.17	[22]
Ethiopia	1100	0-175	3.22	1.10	0.41-2.70	[4,39,43-47]
France	11	0.033-0.19	0.09	0.07	0.05-0.13	[22]
Ghana	1652	0-126	1.09	0.50	0.25-0.98	[4,48-50]
Guatemala	19	0.05-1.48	0.39	0.15	0.05-0.52	[3]
Honduras	55	0.05-7.5	1.62	0.30	0.20-2.55	[51]
Hungary	1	0.15-0.15	0.15	0.15	0.15-0.15	[3]
India	12,983	0-65	0.82	0.46	0.20-0.94	[3,39,52-56]
Indonesia	268	0-4.2	1.01	1.00	0.60-1.30	[3,57,58]
Iran	12	0.1-0.8	0.32	0.20	0.14-0.50	[3]
Ireland	68	0.17-1.36	0.25	0.20	0.20-0.20	[22]
Israel	1	0.275-0.275	0.28	0.28	0.28-0.28	[3]
Italy	2558	0-9	0.26	0.15	0.06-0.28	[22]
Japan	4	0.03-0.1	0.07	0.07	0.04-0.09	[3]
Jordan	6	0.2-1.78	0.56	0.34	0.22-0.48	[3]
Kenya	188	0.1-40	3.10	1.45	0.73-3.75	[3,4,59]
Lithuania	5	0.14-1.1	0.64	0.62	0.56-0.79	[3]
Madagascar	49	0.1-1.2	0.34	0.20	0.20-0.40	[39]
Malawi	374	0-10.8	2.88	2.30	1.20-4.10	[39,60]
Mali	154	0-1.5	0.35	0.20	0.10-0.40	[3,4,39]
Marshall Islands	4	0.2-0.2	0.20	0.20	0.20-0.20	[33]
Mexico	1875	0-29.6	1.37	0.73	0.33-1.59	[3,33,61-63]
Micronesia	6	0-0.6	0.17	0.10	0.00-0.20	[33]
Morocco	4	0.11-0.59	0.35	0.35	0.12-0.58	[3]

Mozambique	192	0-9.9	2.18	1.50	0.80-2.55	[39]
Myanmar	55	0.01-0.35	0.16	0.14	0.10-0.20	[64]
Nepal	19	0.1-1.5	0.62	0.60	0.35-0.90	[39]
New Zealand	125	0.006-5.2	0.12	0.06	0.03-0.11	[3,65]
Nicaragua	16	0.28-1.1	0.63	0.58	0.42-0.77	[10]
Niger	128	0-10.1	0.84	0.40	0.30-0.90	[4,39]
Nigeria	37	0.12-10.3	2.47	1.78	1.17-3.73	[66]
Norway	1342	0.005-8.26	0.71	0.28	0.08-0.99	[3,67-69]
Pakistan	1174	0-30.25	1.89	0.98	0.40-2.68	[3,70-72]
Palau	1	0-0	0.00	0.00	0.00-0.00	[33]
Palestine	12	0.02-0.56	0.18	0.14	0.09-0.23	[73]
Poland	1141	0.05-4.92	0.19	0.11	0.10-0.19	[3,22]
Portugal	449	0-20	0.47	0.20	0.20-0.30	[10]
Russia	1	0.005-0.005	0.01	0.01	0.01-0.01	[68]
Rwanda	17	0.04-0.45	0.23	0.18	0.13-0.33	[39]
Senegal	672	0.05-7.5	1.54	1.00	0.40-2.50	[3,74]
South Africa	43,781	0.03-1140	0.91	0.47	0.22-0.93	[75,76]
South Sudan	3	0.02-0.23	0.10	0.05	0.04-0.14	[4]
Spain	17	0.01-0.35	0.18	0.20	0.14-0.24	[3,10]
Sudan	9	0.4-4.6	2.49	2.10	1.70-3.80	[4]
Sweden	15,176	0-54.22	0.96	0.60	0.28-1.30	[3,22,77]
Switzerland	42	0.029-0.41	0.10	0.07	0.06-0.13	[22]
Tanzania	49	0.16-17.45	2.68	1.55	0.80-3.33	[3,49]
Thailand	1	0.11-0.11	0.11	0.11	0.11-0.11	[3]
Tunisia	4	0.1-0.6	0.30	0.25	0.15-0.45	[3]
Turkey	1	0.4-0.4	0.40	0.40	0.40-0.40	[3]
Uganda	321	0.01-10	0.95	0.60	0.37-1.10	[4,39]
UK	453	0.025-18	0.32	0.10	0.06-0.20	[3,22,78]
Uruguay	1	0.2-0.2	0.20	0.20	0.20-0.20	[3]
USA	218,760	0-22,200	0.97	0.20	0.08-0.50	[33,79,80]
**TOTAL**	**402,452**	**0-22,200**	**0.97**	**0.30**	**0.10-0.70**	

Supplementary Table 2: Global spatially continuous environmental parameters considered in the analysis and prediction of high fluoride concentrations in groundwater. All parameter values are continuous, except where noted as being categorical (CAT). Subsoil refers here to 200 cm depth. The 12 variables used in the final random forest model are highlighted in bold. (At the equator, 30” is approximately equal to 1 km.)

Parameter	Resolution	Parameter	Resolution
Climate		Soil	
**Actual evapotranspiration (AET)[81]**	**30”**	**pH, measured in water, subsoil[82]**	**7.5”**
**Aridity (PET[83]/precipitation[84])**	**30”**	**Silt (0.0002-0.05 mm), weight %, subsoil [82]**	**7.5”**
**Potential evapotranspiration (PET)[83]**	**30”**	Acrisols probability[82]	7.5”
**Precipitation[84]**	**30”**	Alisols probability[82]	7.5”
**Priestley-Taylor alpha coefficient (AET/PET)[81]**	**30”**	Andosols probability, haplic[82]	7.5”
**Temperature[84]**	**30”**	Arenosols probability[82]	7.5”
Geology		Calcisols probability[82]	7.5”
**Acidic igneous rocks (CAT)[85]**	**7.5”**	Cation exchange capacity[82]	7.5”
Active faults, within 0.5 degrees[86]	polygon	Clay (<0.0002 mm), weight %, subsoil [82]	7.5”
Basic igneous rocks (CAT)[85]	7.5”	Coarse fragments (>2 mm), vol. %, subsoil [82]	7.5”
Lithology classes (CAT)[85]	7.5”	FAO soil classes (CAT)[82]	7.5”
Topography		Fine earth bulk density, subsoil [87]	7.5”
**Elevation[88]**	**7.5”**	Fluvisols probability[82]	7.5”
**Maximum multiscale roughness[89]**	**7.5”**	Gleysols probability[82]	7.5”
**Valley bottom flatness[88]**	**7.5”**	Gypsisols probability[82]	7.5”
Downslope curvature[88]	7.5”	Histosols probability, calcic and fibric [82]	7.5”
Compound topographic index[89]	7.5”	Hydrologic soil groups (CAT)[90]	7.5”
Flow accumulation[91]	30”	Nitrogen content, subsoil [82]	7.5”
Geomorphometric classes (CAT)[89]	7.5”	Organic carbon density, subsoil[82]	7.5”
Landform classes (CAT)[85]	7.5”	Organic carbon stock loss(kg/m^2), 0-30 cm, 2001-2015 [92]	7.5”
Maximum multiscale deviation[89]	7.5”	Organic carbon volume, subsoil[82]	7.5”
Convergence index[89]	7.5”	Regosols probability[82]	7.5”
Mountains, low and high (CAT)[85]	7.5”	Sand (0.05-2 mm), weight %, subsoil [82]	7.5”
Profile curvature[89]	7.5”	Soil and sediment thickness[93]	30”
Roughness[89]	7.5”	Solonchaks probability[82]	7.5”
Scale of the maximum multiscale roughness[89]	7.5”	Solonetz probability[82]	7.5”
Slope[94]	7.5”	USDA soil texture classes, subsoil (CAT)[95]	7.5”
Tangential curvature[89]	7.5”	Water content (volumetric %) for 33kPa and 1500kPa, subsoil[96]	7.5”
Terrain ruggedness index[89]	7.5”	Water capacity until wilting points (volumetric %), subsoil[82]	7.5”
Curvature[88]	7.5”	Other	
Vector ruggedness measure[89]	7.5”	Land cover (CAT)[97]	15”

Supplementary Table 3: Fluoride hazard areas and at-risk groundwater-consuming populations by continent. Different calculations were performed that produce a range of values. The “low” estimate counts the groundwater-consuming population only in model cells with >50% probability of fluoride concentrations >1.5 mg/L. The “high” estimate multiplies the entire groundwater-consuming population by the model probability. An intermediate hybrid approach was taken that consists of multiplying the model probability and the groundwater-consuming population of all cells with a probability >25% (see main text for details).

	**Hybrid approach:** (Population in areas above 0.25 threshold x hazard)			**Low estimate:** (Population in areas above 0.5 threshold)			**High estimate:** (Entire population x hazard)		
	At-risk population (% of global total affected)	% area of continent in calc.	% of pop. in continent	At-risk population (% of global total affected)	% area of continent in calc.	% of pop. in continent	At-risk population (% of global total affected)	% area of continent in calc.	% of pop. in continent
Africa	81,886,000 (45.8%)	66%	6.5%	28,197,000 (44.8%)	15%	2.2%	120,725,000 (36.6%)	100%	9.6%
Asia	90,841,000 (50.8%)	51%	2.0%	32,587,000 (51.7%)	2%	0.7%	195,443,000 (59.2%)	100%	4.3%
Australia/ Oceania	6,000 (<0.01%)	41%	<0.01%	1,000 (<0.01%)	8%	<0.01%	102,000 (0.03%)	100%	0.3%
Europe	2,027,000 (1.1%)	18%	0.3%	182,000 (0.3%)	<1%	0.03%	6,260,000 (1.9%)	100%	0.9%
North America	1,773,000 (1.0%)	9%	0.3%	888,000 (1.4%)	2%	0.2%	4,066,000 (1.2%)	100%	0.7%
South America	2,227,000 (1.2%)	36%	0.5%	1,130,000 (1.8%)	8%	0.3%	3,596,000 (1.1%)	100%	0.9%
TOTAL	178,760,000 (100%)	31%	2.4%	62,985,000 (100%)	5%	0.8%	330,192,000 (100%)	100%	4.4%

Supplementary Table 4: Summarized statistics of 100 cross validations of random forests, each containing 1001 trees and using a 80%/20% training/testing split stratified by class. Each tree was grown with samples balanced evenly by class. The test datasets were not balanced, except for the calculation of kappa (repeated ten times per random forest and averaged). The accuracy for different depth ranges of test data was calculated based on the cut-off where sensitivity equals specificity for each depth range.

	**Mean**	**Std. dev.**	**Median**	**Range**
**AUC**	0.9009	0.0016	0.9010	0.8969 - 0.9046
**Kappa**	0.6443	0.0044	0.6443	0.6339 - 0.6546
**Cut-off where sensitivity & specificity equal**	0.5146	0.0046	0.5145	0.5025 - 0.5250
**Accuracy where sensitivity & specificity equal**	0.8221	0.0021	0.8220	0.8178 - 0.8268
**Cut-off at maximum sensitivity + specificity**	0.5001	0.0184	0.5000	0.45 - 0.55
**Accuracy at maximum sensitivity + specificity**	0.8149	0.0093	0.8156	0.7892 - 0.8382
**Accuracy for test measurements between 0-50m depth (n=106,526)**	0.8253	0.0054	0.8256	0.8128 - 0.8383
**Accuracy for test measurements between 50-200m depth (n=62,742)**	0.8161	0.0048	0.8158	0.8027 - 0.8286
**Accuracy for test measurements between 200-600m depth (n=42,310)**	0.8371	0.0066	0.8372	0.8199 - 0.8575
**Accuracy for test measurements >600m depth (n=42,530)**	0.7836	0.0060	0.7838	0.7694 - 0.7987

Supplementary Table 5: Comparison of model predictions and performance of the global model across all of the continents. The mean model probability is based on all of the map pixels in a continent, whereas the mean balanced accuracy (where sensitivity equals specificity) and AUC were calculated on the test data points located within a continent.

Continent	Mean model probability	Mean AUC	Mean balanced acc. ± std dev.	Proportion of points in dataset	Proportion of data points >1.5 mg/L
Africa	0.32 ± 0.16	0.90	0.83 ± 0.01	12%	14%
Asia	0.18 ± 0.13	0.86	0.78 ± 0.01	6%	12%
Australia	0.23 ± 0.18	0.91	0.83 ± 0.01	15%	9%
Europe	0.13 ± 0.11	0.83	0.75 ± 0.01	5%	14%
North America	0.11 ± 0.11	0.90	0.83 ± 0.00	60%	9%
South America	0.22 ± 0.17	0.87	0.81 ± 0.01	1%	42%
GLOBAL	0.20 ± 0.16	0.90	0.82 ± 0.00	100%	10%

Supplementary Table 6: Descriptive statistics of other parameters measured in situ from the data sources listed in Supplementary Table 1. IQR stands for interquartile range.

Parameter	Count	Mean	Median	IQR
Al (µg/L)	97,504	296	40	88
Alkalinity (HCO3) (mg/L)	209,174	421	262	298
As (µg/L)	74,604	21	1	4
B (mg/L)	79,742	2.53	0.10	0.31
Ba (µg/L)	43,081	305	48	83
Br (µg/L)	68,705	3180	55	122
Ca (mg/L)	241,968	80	43	71
Cd (µg/L)	20,894	279	0.07	0.48
Cl (mg/L)	325,199	354	16	72
Co (µg/L)	18,552	31	0.28	2.06
Cr (µg/L)	50,734	14	0	2
Cu (µg/L)	42,552	88	4	14
Depth (m)	255,332	306	70	311
EC (µS/cm)	302,756	1528	551	941
Fe (mg/L)	166,036	15	0.06	0.46
Hg (µg/L)	10,936	4.16	0.10	0.25
I (µg/L)	9,377	1031	10	48
K (mg/L)	220,856	9.16	2.61	4.51
Li (µg/L)	31,844	196	18	45
Mg (mg/L)	277,891	39	12	28
Mn (µg/L)	181,592	334	40	128
Mo (µg/L)	24,458	32	2.26	6.85
Na (mg/L)	294,287	245	35	152
NH4 (mg/L)	20,609	0.52	0.05	0.11
Ni (µg/L)	24,955	31	1.40	3.09
NO2-N (µg/L)	29,316	123	50	0.00
NO3 (mg/L)	198,048	20	2.22	7.06
O2 (mg/L)	51,450	3.66	3.00	5.80
P (mg/L)	30,410	1.88	0.02	0.06
Pb (µg/L)	27,247	17	0.51	2.92
pH	318,596	7.43	7.50	0.87
PO4 (mg/L)	21,016	0.24	0.05	0.06
S (mg/L)	941	121	24.2	60.7
Sb (µg/L)	12,838	6.29	0.10	0.45

Si (mg/L)	4,498	143	7.41	21
SO4 (mg/L)	249,798	198	29	115
Sr (µg/L)	45,318	2595	310	750
TDS (mg/L)	219,002	1191	441	734
Temperature (°C)	190,622	17	16	9.08
U (µg/L)	83,954	12	0.10	0.67
V (µg/L)	83,016	1	0.20	1.75
Zn (µg/L)	55,420	1492	18	50

References in Supplementary Materials

1 Houben, G., Tünnermeier, T., Eqrar, N. & Himmelsbach, T. Hydrogeology of the Kabul Basin (Afghanistan), part II: groundwater geochemistry. *Hydrogeology journal* **17**, 935-948 (2009).
2 Broshears, R. E., Akbari, M. A., Chornack, M. P., Mueller, D. K. & Ruddy, B. C. Inventory of ground-water resources in the Kabul Basin, Afghanistan. (U. S. Geological Survey, 2005).
3 UNEP. Water Quality, 2005 State of the UNEP GEMS/Water Global Network and Annual Report. (2005).
4 UNHCR. Borehole GIS Portal. *http://wash.unhcr.org/wash-gis-portal/*, doi:http://wash.unhcr.org/wash-gis-portal/ (2019).
5 Nicolli, H. B., Suriano, J. M., Peral, M. A. G., Ferpozzi, L. H. & Baleani, O. A. Groundwater contamination with arsenic and other trace elements in an area of the Pampa, Province of Córdoba, Argentina. *Environmental Geology and Water Sciences* **14**, 3-16 (1989).
6 Blarasin, M., Cabrera, A. & Matteoda, E. in *XXXIII IAH -. 7º ALHSUD Congress* (Zacatecas, Mexico, 2004).
7 Zabala, M. E., Manzano, M. & Vives, L. Assessment of processes controlling the regional distribution of fluoride and arsenic in groundwater of the Pampeano Aquifer in the Del Azul Creek basin (Argentina). *Journal of hydrology* **541**, 1067-1087 (2016).
8 ACUMAR. BDH-CMR. Base de Datos Hidrológicos de la Cuenca Matanza- Riachuelo. *http://www.bdh.acumar.gov.ar/bdh3/index_contenido.php?xgap_historial=reset* (accessed 11.11.2020).
9 Smedley, P., Nicolli, H., Macdonald, D., Barros, A. & Tullio, J. Hydrogeochemistry of arsenic and other inorganic constituents in groundwaters from La Pampa, Argentina. *Applied Geochemistry* **17**, 259-284 (2002).
10 Morgada, M. E., Mateu, M., Bundschuh, J. & Litter, M. I. Arsenic in the Iberoamerican region. The IBEROARSEN Network and a possible economic solution for arsenic removal in isolated rural zones. *e-Terra* **5**, 1-11 (2008).
11 Western Austalia Department of Water and Environmental Regulation. Water INformation (WIN) database - discrete sample data. *https://www.water.wa.gov.au/water-topics/water-quality/monitoring-and-assessing-water-quality* (accessed 03.11.2020).
12 State of Queensland, Department of Natural Resources,, Mines and Energy. Groundwater Database - Queensland. *http://qldspatial.information.qld.gov.au/catalogue/custom/search.page?q=%22Groundwater%20Database%20-%20Queensland* (accessed 01.11.2020).
13 Government of South Australia. WaterConnect, Enviro Data SA. *https://www.waterconnect.sa.gov.au/Pages/Advanced-Map-Search.aspx* (accessed 04.11.2020).
14 Victoria State Government, Department of Environment, Land, Water and Planning. Water Measurement Information System (WMIS). *https://data.water.vic.gov.au/* (accessed 12.11.2020).
15 Northern Territory Government, Environment and Natural Resources Department. Northern Territory bore locations, water quality and groundwater levels. *https://data.nt.gov.au/dataset/nt-bore-locations-water-quality-and-groundwater-levels/resource/7c89cd4d-5d14-4599-8c6e-345a9255be67* (accessed 14.12.2020).
16 Ivkovic, K., Watkins, K., Cresswell, R. & Bauld, J. A groundwater quality assessment of the fractured rock aquifers of the Piccadilly Valley, South Australia. http://hdl.handle.net/102.100.100/217514?index=1 (1998).
17 Fitzgerald, J. *et al.* Groundwater quality and environmental health implications. *Anangu Pitjantjara Lands, South Australia, A Report from Bureau of Rural Sciences*, 1-30 (1999).
18 Larsen, R. M. *A Groundwater Quality Assessment of the Jandakot Mound, Swan Coastal Plain, Western Australia*. (Australian Geological Survey Organisation, 1998).
19 NSW Office of Water. NSW Office of Water - Groundwater quality extract. Bioregional Assessment Source Dataset. *http://data.bioregionalassessments.gov.au/dataset/74da836a-7b97-4278-9034-c9a259a34fbb* (accessed 10.12.2018).
20 Hoque, M., McArthur, J., Sikdar, P., Ball, J. & Molla, T. Tracing recharge to aquifers beneath an Asian megacity with Cl/Br and stable isotopes: the example of Dhaka, Bangladesh. *Hydrogeology journal* **22**, 1549-1560 (2014).
21 Kinniburgh, D. & Smedley, P. Arsenic contamination of groundwater in Bangladesh. https://www2.bgs.ac.uk/groundwater/health/arsenic/Bangladesh/home.html.
22 EEA. Waterbase - Water Quality. *https://www.eea.europa.eu/data-and-maps/data/waterbase-water-quality-2* (2019).
23 MMEWR & (Ministry of Minerals, E. a. W. R. (eds Botswana & Department of Water Affairs) 27 (Gaborone, Botswana, 2004).

24 Ezaki, S., Pérez-Aguilar, A., Hypolito, R. & Shinzato, M. C. Anomalias de flúor nas águas subterrâneas do estado de São Paulo. *Revista do Instituto Geológico, São Paulo* **37**, 65-98 (2016).
25 Geological Survey of Brazil. http://siagasweb.cprm.gov.br ; http://rimasweb.cprm.gov.br (accessed 24.03.2015).
26 Bretzler, A. *et al.* Groundwater arsenic contamination in Burkina Faso, West Africa: Predicting and verifying regions at risk. *Science of the Total Environment* **584**, 958-970 (2017).
27 Ministry of Rural Development of Cambodia. Cambodia WellMap. http://www.cambodiawellmap.com/ (2010).
28 Fantong, W. Y. *et al.* Geochemical provenance and spatial distribution of fluoride in groundwater of Mayo Tsanaga River Basin, Far North Region, Cameroon: implications for incidence of fluorosis and optimal consumption dose. *Environmental geochemistry and health* **32**, 147-163 (2010).
29 Boyle, D. R., Spirito, W. A. & Adcock, S. W. Groundwater hydrogeochemical survey of central New Brunswick. 125 (1996).
30 Environment and Climate Change Canada. Groundwater-AssessmentArea-WaterQuality-2010-v1.csv. https://open.canada.ca (2010).
31 Government of Alberta. Domestic Well Water Quality in Alberta - Routine Chemistry. https://open.canada.ca/data/en/dataset/44dc425c-535f-4dbc-98f7-09fc772a6e95 (accessed 13.11.2020).
32 Ontario Ministry of Environment, Conservation and Parks,. Provincial Groundwater Monitoring Network (PGMN). https://data.ontario.ca/dataset/provincial-groundwater-monitoring-network (accessed 16.11.2020).
33 Read, E. K. *et al.* Water quality data for national-scale aquatic research: The Water Quality Portal. *Water Resources Research* **53**, 1735-1745 (2017).
34 Smedley, P., Zhang, M., Zhang, G. & Luo, Z. Mobilisation of arsenic and other trace elements in fluviolacustrine aquifers of the Huhhot Basin, Inner Mongolia. *Applied Geochemistry* **18**, 1453-1477 (2003).
35 Currell, M., Cartwright, I., Raveggi, M. & Han, D. Controls on elevated fluoride and arsenic concentrations in groundwater from the Yuncheng Basin, China. *Applied Geochemistry* **26**, 540-552 (2011).
36 Li, J. *et al.* Paleo-environment from isotopes and hydrochemistry of groundwater in East Junggar Basin, Northwest China. *Journal of Hydrology* **529**, 650-661 (2015).
37 Su, H., Wang, J. & Liu, J. Geochemical factors controlling the occurrence of high-fluoride groundwater in the western region of the Ordos basin, northwestern China. *Environmental pollution* **252**, 1154-1162 (2019).
38 Zhang, X., Gao, X., Li, C., Luo, X. & Wang, Y. Fluoride contributes to the shaping of microbial community in high fluoride groundwater in Qiji County, Yuncheng City, China. *Scientific reports* **9**, 1-10 (2019).
39 Charity Water. Water Quality Database. New York, USA (2020).
40 Abdel-Azeem, A. M. *et al.* in *Water Technol. Conf.* 1491-1513.
41 Srikanth, R., Viswanatham, K., Kahsai, F., Fisahatsion, A. & Asmellash, M. Fluoride in groundwater in selected villages in Eritrea (North East Africa). *Environmental monitoring and assessment* **75**, 169-177 (2002).
42 Haile, E. Groundwater chemistry and recharge rate in crystalline rocks: case study from the Eritran highland. *(thesis) KTH, School of Architecture and the Built Environment* (2005).
43 Groen, J., Kebede, S., Ketema, A., Kleinendorst, T. & Asrat, A. Acacia Data, GW4E Groundwater mapping for climate resilient WASH in arid and semi-arid areas of Ethiopia, final report Phase 2. https://gw4e.acaciadata.com/home (2020).
44 Ministry of Water, Irrigation and Electricity of Ethiopia. Acacia Data, MOWIE. https://gw4e.acaciadata.com/home (accessed 01.10.2020).
45 Rango, T., Bianchini, G., Beccaluva, L. & Tassinari, R. Geochemistry and water quality assessment of central Main Ethiopian Rift natural waters with emphasis on source and occurrence of fluoride and arsenic. *Journal of African Earth Sciences* **57**, 479-491 (2010).
46 Reimann, C., Bjorvatn, K., Tekle-Haimanot, R., Melako, Z. & Siewers, U. Drinking water quality, Rift Valley, Ethiopia. *Norges geologiske undersøkelse, Report* **2002**, 132, https://hdl.handle.net/11250/2665168 (2002).
47 Bretzler, A. *et al.* Groundwater origin and flow dynamics in active rift systems–A multi-isotope approach in the Main Ethiopian Rift. *Journal of hydrology* **402**, 274-289 (2011).
48 Smedley, P. L. Arsenic in rural groundwater in Ghana: part special issue: hydrogeochemical studies in sub-Saharan Africa. *Journal of African Earth Sciences* **22**, 459-470 (1996).
49 Smedley, P. *et al.* Fluoride in groundwater from high-fluoride areas of Ghana and Tanzania. British Geological Survey, Commissioned Report CR/02/316 (2002).
50 Deal, P. T. & Sabatini, D. A. Utilizing indicator kriging to identify suitable zones for manual drilling in weathered crystalline basement aquifers. *Groundwater for Sustainable Development*, 11, 100402 (2020).

51 Foletti, C. Diagnostico de fluorosis dental en 39 comunidades del Valle de Sula, Honduras. (Swiss Agency for Development Cooperation (SDC) and Programa de Saneamiento de la Secretaria de Salud (SdS), 1991).
52 Gupta, S., Deshpande, R., Agarwal, M. & Raval, B. Origin of high fluoride in groundwater in the North Gujarat-Cambay region, India. *Hydrogeology Journal* **13**, 596-605 (2004).
53 Jha, B. Ground water quality in shallow aquifers of India. *Central ground water board, Ministry of water resources, Govt. of India* https://www.indiawaterportal.org/sites/default/files/iwp2/Groundwater_Quality_Shallow_Aquifers_India_CGWB_2010.pdf (2010).
54 Gupta, S. Annual Report 2013-14. (Government of India Central Ground Water Board, Faridabad, India, 2015) http://cgwb.gov.in/Annual-Reports/Annual%20Report-2013-14.pdf .
55 Hazarika, S. & Bhuyan, B. Fluoride, arsenic and iron content of groundwater around six selected tea gardens of Lakhimpur District, Assam, India. *Arch Appl Sci Res* **5**, 57-61 (2013).
56 McArthur, J., Sikdar, P., Leng, M., Ghosal, U. & Sen, I. Groundwater quality beneath an Asian megacity on a delta: Kolkata's (Calcutta's) disappearing arsenic and present manganese. *Environmental science & technology* **52**, 5161-5172 (2018).
57 Heikens, A. *et al.* The impact of the hyperacid Ijen Crater Lake: risks of excess fluoride to human health. *Science of the Total Environment* **346**, 56-69 (2005).
58 Marohn, C., Distel, A., Tomlinson, R., Noordwijk, M. v. & Cadisch, G. Impacts of soil and groundwater salinization on tree crop performance in post-tsunami Aceh Barat, Indonesia. *Natural Hazards and Earth System Sciences* **12**, 2879 (2012).
59 Näslund, J. & Snell, I. GIS-mapping of fluoride contaminated groundwater in Nakuru and Baringo district, Kenya (Master Thesis), Lulea University of Technology, Sweden, 2005. Retrieved from http://urn.kb.se/resolve?urn=urn:nbn:se:ltu:diva-56091, 2005).
60 Pritchard, M., Mkandawire, T. & O'neill, J. Assessment of groundwater quality in shallow wells within the southern districts of Malawi. *Physics and Chemistry of the Earth, Parts A/B/C* **33**, 812-823 (2008).
61 Knappett, P. S. *et al.* Rising arsenic concentrations from dewatering a geothermally influenced aquifer in central Mexico. *Water Research* **185**, 116257 (2020).
62 INCA. *Arsénico y fluoruro en agua: riesgos y perspectivas desde la sociedad civil y la academia en México*. (2018).
63 Planer-Friedrich, B. Hydrogeological and hydrochemical investigations in the Rioverde basin, Mexico. *Freiberg Online Geosciences*,Vol. 3, doi:10.23689/fidgeo-871 (2000).
64 Van Geen, A. *et al.* Confirmation of elevated arsenic levels in groundwater of Myanmar. *Science of the Total Environment* **478**, 21-24 (2014).
65 Daughney, C. J. & Reeves, R. R. Definition of hydrochemical facies in the New Zealand national groundwater monitoring programme. *Journal of Hydrology (New Zealand)* **44**, 105-130 (2005).
66 Dibal, H., Schoeneich, K., Garba, I., Lar, U. & Bala, E. Occurrence of fluoride in the drinking waters of Langtang area, north central Nigeria, http://hdl.handle.net/123456789/578 (2012).
67 Banks, D., Frengstad, B., Midtgård, A. K., Krog, J. R. & Strand, T. The chemistry of Norwegian groundwaters: I. The distribution of radon, major and minor elements in 1604 crystalline bedrock groundwaters. *Science of the Total environment* **222**, 71-91 (1998).
68 de Caritat, P., Danilova, S., Reimann, C. & Storrø, G. Groundwater composition near the nickel—copper smelting industry on the Kola Peninsula, central Barents Region (NW Russia and NE Norway). *Journal of Hydrology* **208**, 92-107 (1998).
69 Frengstad, B., Skrede, A. K. M., Banks, D., Krog, J. R. & Siewers, U. The chemistry of Norwegian groundwaters: III. The distribution of trace elements in 476 crystalline bedrock groundwaters, as analysed by ICP-MS techniques. *Science of the Total Environment* **246**, 21-40 (2000).
70 Podgorski, J. E. *et al.* Extensive arsenic contamination in high-pH unconfined aquifers in the Indus Valley. *Science Advances* **3**, doi:10.1126/sciadv.1700935 (2017).
71 Rafique, T. *Occurrence, Distribution and origin of Fluoride-Rich Groundwater in the Thar Desert, Pakistan*, University of Sindh Jamshoro, http://173.208.131.244:9060/xmlui/handle/123456789/8673 (2008).
72 Ali, W. *et al.* Elucidating various geochemical mechanisms drive fluoride contamination in unconfined aquifers along the major rivers in Sindh and Punjab, Pakistan. *Environmental Pollution* **249**, 535-549 (2019).
73 Qannam, Z. A hydrogeological, hydrochemical and environmental study in Wadi Al Arroub drainage basin, south west Bank, Palestine. *Freiberg Online Geosciences*, Vol.9, https://doi.org/10.23689/fidgeo-878 (2003).
74 Service de Gestion et de Planification des Ressources en Eau (SGPRE). Synthèse des données géochimiques. Interprétations en terme de modèle conceptuel des écoulements. (République du Sénégal, Ministère de l'Energie et de l'Hydraulique, 2001).

75 McCaffrey, L. & Willis, J. *Distribution of fluoride-rich groundwater in the eastern and Mogwase regions of the Northern and North-West Provinces*. Water Research Commission South Africa, WRC Report No. 526/1/01, https://www.wrc.org.za/wp-content/uploads/mdocs/526-1-01.pdf (2001).
76 South Africa Department of Water Affairs and Forestry. National Groundwater Information System (NGIS). *http://www.dwa.gov.za/Groundwater/NGIS.aspx* (2020).
77 Geological Survey of Sweden. Data from Environmental Monitoring of Groundwater. *https://www.sgu.se/produkter/geologiska-data/oppna-data/grundvatten-oppna-data/miljoovervakning-av-grundvatten/* (2007).
78 UK Environment Agency. Water Quality Archive (Beta). *https://environment.data.gov.uk/water-quality/view/download* (accessed 15.01.2021).
79 U.S. Geological Survey. National Uranium Resource Evaluation (NURE) Hydrogeochemical and Stream Sediment Reconnaissance data. *https://mrdata.usgs.gov/nure/water/* (2004).
80 Texas Water Development Board. Groundwater Database (GWDB). *http://www.twdb.texas.gov/groundwater/data/gwdbrpt.asp* (accessed 30.11.2019).
81 Trabucco, A. & Zomer, R. Global soil water balance geospatial database. *CGIAR Consortium for Spatial Information, Published online, available from the CGIAR-CSI GeoPortal at: http://www. cgiar-csi. org (last access: January 2013)* (2010).
82 Hengl, T. *et al.* SoilGrids250m: Global gridded soil information based on machine learning. *PLoS one* **12**, e0169748 (2017).
83 Trabucco, A. & Zomer, R. J. Global aridity index (global-aridity) and global potential evapo-transpiration (global-PET) geospatial database. *CGIAR Consortium for Spatial Information* (2009).
84 Fick Stephen, E. & Hijmans Robert, J. W. 2: new 1-km spatial resolution climate surfaces for global land areas. *International Journal of Climatology* (2017).
85 Hengl, T. Global landform and lithology class at 250 m based on the USGS global ecosystem map (Version 1.0). *http://doi.org/10.5281/zenodo.1464846*, doi:http://doi.org/10.5281/zenodo.1464846 (2018).
86 Styron, R. & Pagani, M. The GEM global active faults database. *Earthquake Spectra* **36**, 160-180 (2020).
87 Hengl, T. Soil bulk density (fine earth) 10 x kg / m-cubic at 6 standard depths (0, 10, 30, 60, 100 and 200 cm) at 250 m resolution. *http://doi.org/10.5281/zenodo.2525665*, doi:http://doi.org/10.5281/zenodo.2525665 (2018).
88 Hengl, T. Global DEM derivatives at 250 m, 1 km and 2 km based on the MERIT DEM (Version 1.0). *http://doi.org/10.5281/zenodo.1447210*, doi:http://doi.org/10.5281/zenodo.1447210 (2018).
89 Amatulli, G., McInerney, D., Sethi, T., Strobl, P. & Domisch, S. Geomorpho90m-Global high-resolution geomorphometry layers: empirical evaluation and accuracy assessment. Report No. 2167-9843, (PeerJ Preprints, 2019).
90 Ross, C. W. *et al.* HYSOGs250m, global gridded hydrologic soil groups for curve-number-based runoff modeling. *Scientific data* **5**, 180091 (2018).
91 Lehner, B., Verdin, K. & Jarvis, A. Available at http://hydrosheds.cr.usgs.gov (World Wildlife Fund US, Washington, DC., 2006).
92 Wheeler, I. & Hengl, T. Soil organic carbon stock (0–30 cm) in kg/m2 time-series 2001–2015 based on the land cover changes. *http://doi.org/10.5281/zenodo.2529721*, doi:http://doi.org/10.5281/zenodo.2529721 (2018).
93 Pelletier, J. *et al.* Global 1-km gridded thickness of soil, regolith, and sedimentary deposit layers. *ORNL DAAC* (2016).
94 Yamazaki, D. *et al.* A high-accuracy map of global terrain elevations. *Geophysical Research Letters* **44**, 5844-5853 (2017).
95 Hengl, T. Soil texture classes (USDA system) for 6 soil depths (0, 10, 30, 60, 100 and 200 cm) at 250 m. *http://doi.org/10.5281/zenodo.2525817*, doi:http://doi.org/10.5281/zenodo.2525817 (2018).
96 Hengl, T. & Gupta, S. Soil water content (volumetric %) for 33kPa and 1500kPa suctions predicted at 6 standard depths (0, 10, 30, 60, 100 and 200 cm) at 250 m resolution *http://doi.org/10.5281/zenodo.1447210*, doi:http://doi.org/10.5281/zenodo (2019).
97 Friedl, M. A. *et al.* MODIS Collection 5 global land cover: Algorithm refinements and characterization of new datasets. *Remote sensing of Environment* **114**, 168-182 (2010).
