# Supplementary material for: Global analysis and prediction of fluoride in groundwater
Source: Nat Commun. 2022 Aug 1;13:4232. doi: 10.1038/s41467-022-31940-x (PMC9343638; doi:10.1038/s41467-022-31940-x)
Supplement: Supplementary file 2 — Description of Additional Supplementary Files [file 41467_2022_31940_MOESM2_ESM.pdf]

File name: Supplementary Data 1

Description: The files in code.zip include the R scripts for performing the modelling as well as a sample data file of publicly available measurements of fluoride in groundwater.
